# Comparative Transcriptomic, Anatomical and Phytohormone Analyses Provide New Insights Into Hormone-Mediated Tetraploid Dwarfing in Hybrid Sweetgum (*Liquidambar styraciflua × L. formosana*)

**DOI:** 10.3389/fpls.2022.924044

**Published:** 2022-06-27

**Authors:** Siyuan Chen, Yan Zhang, Ting Zhang, Dingju Zhan, Zhenwu Pang, Jian Zhao, Jinfeng Zhang

**Affiliations:** ^1^National Engineering Research Center of Tree Breeding and Ecological Restoration, Key Laboratory of Genetics and Breeding in Forest Trees and Ornamental Plants, Ministry of Education, The Tree and Ornamental Plant Breeding and Biotechnology Laboratory of National Forestry and Grassland Administration, College of Biological Sciences and Biotechnology, Beijing Forestry University, Beijing, China; ^2^College of Landscape Architecture, Beijing University of Agriculture, Beijing, China; ^3^Guangxi Bagui Forest and Flowers Seedlings Co., Ltd., Nanning, China

**Keywords:** polyploidization, RNA-seq, dwarf, plant hormone, hybrid sweetgum

## Abstract

Polyploid breeding is an effective approach to improve plant biomass and quality. Both fast growth and dwarf types of *in vitro* or *ex vitro* plants are produced after polyploidization. However, little is known regarding the dwarf type mechanism in polyploids grown *in vitro*. In this study, the morphological and cytological characteristics were measured in tetraploid and diploid hybrid sweetgum (*Liquidambar styraciflua* × *L. formosana*) with the same genetic background. RNA sequencing (RNA-Seq) was used to analyse shoot and root variations between tetraploid and diploid plants; important metabolites were validated. The results showed that the shoot and root lengths were significantly shorter in tetraploids than in diploids after 25 d of culture. Most tetraploid root cells were wider and more irregular, and the length of the meristematic zone was shorter, while tetraploid cells were significantly larger than diploid cells. Differentially expressed genes (DEGs) were significantly enriched in the plant growth and organ elongation pathways, such as plant hormone biosynthesis and signal transduction, sugar and starch metabolism, and cell cycles. Hormone biosynthesis and signal transduction genes, such as *YUCCA*, *TAA1*, *GH3*, *SAUR*, *CPS*, *KO*, *KAO*, *GA20ox*, *GA3ox*, *BAS1* and *CYCD3*, which help to regulate organ elongation, were generally downregulated. The auxin, gibberellin, and brassinolide (BL) contents in roots and stems were significantly lower in tetraploids than in diploids, which may greatly contribute to slow growth in the roots and stems of tetraploid regenerated plants. Exogenous gibberellic acid (GA_3_) and indole-3-acetic acid (IAA), which induced plant cell elongation, could significantly promote growth in the stems and roots of tetraploids. In summary, comparative transcriptomics and metabolite analysis showed that the slow growth of regenerated tetraploid hybrid sweetgum was strongly related to auxin and gibberellin deficiency. Our findings provide insights into the molecular mechanisms that underlie dwarfism in allopolyploid hybrid sweetgum.

## Introduction

Polyploidy or genome doubling results from the merging of more than two full sets of chromosomes (Comai, 2005; Song and Chen, 2015); polyploidization substantially contributes to plant evolution, new species formation, and plant genetic improvement (Leitch and Leitch, 2008; Jiao et al., 2011). After chromosome doubling, polyploid plants often exhibit multiple variations and generally develop large organs, strong resistance, high crop yield, wood quality, and ornamental value (Zhu et al., 1995; Leitch and Leitch, 2008; Tiku et al., 2014). However, after polyploidization, the changes in plant height differ among species; there is usually a fast growing type (Ni et al., 2009; Miller et al., 2012; Dai et al., 2015; Liao et al., 2016) and a dwarf type (Mu et al., 2012; Tokumoto et al., 2016; Xue et al., 2017). Similar to fast growth, dwarfing is a common phenomenon in tetraploid plants, especially woody plants. It remains unclear why specific species exhibit fast or slow growth after chromosome doubling. Dwarf tetraploid plants have a high ornamental value (Ye et al., 2010); they are often favoured in courtyard and urban landscapes where space may be limited. Such plants often exhibit strong resistance to abiotic and biotic stresses (Tan et al., 2015) and can be used as parents for triploid production.

In addition, fast growth (Gantait et al., 2011; Salma et al., 2018) and slow growth (Gu et al., 2005; Rao et al., 2019) polyploids have been observed *in vitro*. Regenerated plants of tetraploid *Lagerstroemia indica* (Tong, 2007), *Ziziphus jujube* (Gu et al., 2005), *Punica granatum* (Shao et al., 2003), and *Lycium ruthenicum* (Rao et al., 2019) exhibit dwarfing or shortened roots, which can affect regeneration efficiency. The transcriptional expression levels of fast and slow-growing polyploids may substantially change after polyploidization (Cheng et al., 2015; Ma et al., 2016). However, there have been few reports (Ma et al., 2016) concerning the molecular mechanism that underlies the *in vitro* dwarf polyploid type. Moreover, to promote stem and root elongation by improving culture conditions or medium formulation, there is a need to understand which genes and metabolites substantially change in tetraploid plants.

Organ elongation is an important process in plant tissue culture. The degree of difficulty in modifying organ length varies among species (Bizet et al., 2015). Light intensity, agar concentration, growth regulators, and nutrients can affect the lengths of adventitious shoots, stem segments, and roots in tissue culture. Plant hormones have critical effects on organ lengths *in vitro*; for example, auxin, gibberellin, and brassinolide (BL) can promote root or stem elongation (Su et al., 2011). Tong (2007) found that a diploid medium was unsuitable for *in vitro* tetraploid plants. After polyploidization, plants exhibit altered hormone synthesis and changes in the levels of metabolites related to signal transduction genes (Mu et al., 2012; Ma et al., 2016).

Forest trees of *Liquidambar* spp. have important economic and ecological value (Harlow et al., 1996; Merkle and Battle, 2000; Zheng et al., 2015). Chinese sweetgum (*Liquidambar formosana* Hance) is mainly used for ornamental and medicinal purposes (Chen et al., 2020). American sweetgum (*Liquidambar styraciflua* L.) is an important feedstock for the timber and paper industries in South America. In a previous study, several clones of hybrid sweetgum demonstrated obvious heterosis in terms of growth rate and wood density (Merkle et al., 2010). For allotetraploid species, the advantages of some traits have been linked to heterosis (Fuentes et al., 2014). Tetraploid hybrid sweetgum (*L. styraciflua* × *L. formosana*; Zhang et al., 2017) has been reported to show dwarf traits (Unpublished). Some new varieties of sweetgum, such as the dwarfing cultivar ‘Gumball’, have a shrubby appearance (maximum height of 4.5 m) (Sommer et al., 1999).

What are the key contributors to the dwarf resulting from genome doubling in hybrid sweetgum? Whether the roots and stems of tetraploid hybrid sweetgum have mutated; whether endogenous hormones in plants, the most important factors affecting plant growth, change their regulatory mechanism after polyploidy; whether the growth of dwarf tetraploid can be promoted by application of exogenous plant growth regulators. To answer those questions, in this study, *in vitro* tetraploid hybrid sweetgum was compared with diploid hybrid sweetgum. Morphological changes in stems and roots were observed at various growth stages; organ variations of tetraploid hybrid sweetgum were analysed at the phenotypic, cytological, and molecular levels. Histological analysis, phytohormone analysis and transcriptome sequencing were also performed to identify mechanisms responsible for the variation.

## Materials and Methods

### Plant Material and Determination of Ploidy Level

Three genotypes (Z1, Z2, and Z3) of diploid hybrid sweetgum (D1, D2, and D3) and their tetraploid counterparts (T1, T2, and T3; same genetic background) were used as research material in the present study. *In vitro* diploid and tetraploid families were obtained by hybridization (*L. styraciflua* × *L. formosana*). Artificial tetraploids were acquired from *in vitro* shoot regeneration *via* colchicine treatment, as previously described (Zhang et al., 2017). To acquire sufficient clones for each genotype, all materials were derived from tissue culture propagation. Plantlets were rooted in half-strength woody plant medium supplemented with 2.0 mg/L indole butyric acid, 0.1 mg/L naphthaleneacetic acid, 2.0 g/L agar, 4.0 g/L polygel, and 30 g/L sucrose, called primitive rooting medium. The pH was adjusted to 5.8 prior to autoclaving. Vigorously growing leaves of all genotypes were collected; the ploidy level was analysed using a Cyflow Ploidy Analyser (Partec, Görlitz, SN, Germany). Ploidy level was determined in accordance with the methods described by Zhang et al. (2017).

### Plant Height Measurement

To maintain consistency, adventitious shoots at the same regeneration time were excised and cut to approximately 1.5 cm for rooting. The rooting medium contained half-strength woody plant medium supplemented with 2.0 mg/L indole butyric acid and 0.1 mg/L naphthaleneacetic acid; it was placed in a culture bottle with 50 ml medium. The phenotypes of the plantlets were measured after 0, 25, 50, and 70 d of subculture: 0–25 d was stage I, 25–50 d was stage II, and 50–70 d was stage III. In addition to plant height, ground diameter, number of stem nodes, root length, number of roots, leaf area, petiole length, and number of leaves were measured. The primary root diameter was measured by ImageJ.^1^ Three biological replicates were performed; each consisted of three genotypes, including three to five plantlets for each genotype.

### Anatomical Analysis

Leaves (second or third leaf position), roots, stems, and shoot tips of diploid and tetraploid plants at 25, 50, and 70 d were prepared as paraffin sections. Materials were immobilised in formaldehyde alcohol acetic acid solution (5 ml 38% formaldehyde +5 ml glacial acetic acid +90 ml 50% ethanol, 1:1:18, by vol.) for 24 h. The materials were dehydrated in ethanol and pellucidum in xylene, then embedded in paraffin. The slices had a thickness of 8 mm; they were stained with saffron and solid green. All materials were observed under a biomicroscope (BX43, Olympus, Tokyo, Japan) and photographed by an image acquisition and analysis system (DP73, Olympus). The following items were observed: the thicknesses of leaves, spongy tissues, palisade tissues, and main veins; stem diameter and the thicknesses of epidermis, cortex, and vascular column; root diameter, epidermis cortex radius, and central column diameter; upper column diameter and cell transection area of leaf xylem, as well as cell transection areas of the lower epidermis, spongy tissue, and palisade tissue; cell transection areas of xylem, cortex, and phloem, as well as stem pith; and cell transection areas of xylem, epidermis, and root cortex. Three biological replicates were obtained. Each section was randomly measured for 20–30 cells, with three to five replicate measurements. All measurements were performed using ImageJ software.

### RNA Extraction

Four types of RNA were extracted (each containing three genotypes): diploid stem (DS), tetraploid stem (TS), diploid root (DR), and tetraploid root (TR). Three biological replicates were used for total RNA extraction. The 12 samples were named DS-1, DS-2, DS-3, TS-1, TS-2, TS-3, DR-1, DR-2, DR-3, TR-1, TR-2, and TR-3, respectively. All materials were collected after 50 d of subculture, immediately frozen in liquid nitrogen, and stored at −80°C until RNA extraction. RNA extraction was performed in accordance with the methods described by Zhang et al. (2019). RNA purity was checked using a NanoPhotometer^®^ spectrophotometer (IMPLEN, CA, United States). RNA concentrations were measured using a Qubit^®^ RNA Assay Kit in a Qubit^®^2.0 fluorometer (Life Technologies, Carlsbad, CA, United States).

### Sequencing and Data Analysis

The construction and sequencing of a cDNA library were performed by Shanghai OE Biotech. Co., Ltd. (China); fragments with lengths of 150–200 bp were selected using an AMPure XP system (Beckman Coulter, Beverly, MA, United States). Library quality was assessed on a bioanalyzer (2100, Agilent, Santa Clara, CA, United States). Nine cDNA libraries were sequenced on a Hiseq 2000 platform (Illumina, CA, United States). High-quality reads were assembled *de novo* using Trinity software (Grabherr et al., 2011). The final unique consensus sequences were regarded as unigenes. For gene annotation, BLAST was used to align the unigenes to the National Center for Biotechnology Information (NCBI) nonredundant protein database (Nr), NCBI non-redundant nucleotide database (Nt), Swiss-Prot protein database (Swiss-Prot), protein families database (Pfam), Kyoto Encyclopaedia of Genes and Genomes (KEGG), and Clusters of Orthologous Groups of proteins (COG). Gene Ontology (GO)^2^ annotation was performed using Blast2GO software (version 2.5; Gotz et al., 2008) based on the results of Nr and Pfam annotation. Fragments per kilobase per million base pairs (FPKM) values were used to evaluate the expression patterns of unigenes with the thresholds of |log_2_Ratio| ≥ 1.0 and false discovery rate ≤ 0.001.

### Analysis of DEGs

GO enrichment analysis (corrected *p* < 0.05) of the DEGs was performed with the GOseq R package after the data had been evaluated using the Kolmogorov–Smirnov test. KEGG pathways were assigned to the unigenes and pathway maps using the online KEGG Automatic Annotation Server^3^ with the bidirectional best-hit method (Moriya et al., 2007). KOBAS software (Mao et al., 2005) was used to determine DEG enrichment in KEGG pathways.

### Gene Clustering and Visualization

Heat maps were used to visualise gene expression; the maps were constructed using log_2_-transformed FPKM values in MeV v4.8.1 (Saeed et al., 2003), with Pearson’s correlation as a similarity metric.

### qPCR Validation and Expression Analysis

Quantitative real-time polymerase chain reaction (qPCR) was conducted in accordance with the methods described by Zhang et al. (2019). Primer sequences are listed in Supplementary Table S1. 18s rRNA was used as the endogenous reference gene; relative expression levels were calculated using the 2^−ΔΔCt^ method (Livak and Schmittgen, 2001).

### Soluble Sugar and Starch Content Measurement

Soluble sugar content was determined by the anthrone colorimetry method (Fukao et al., 2006). Fructose (Fructose assay kit A085-1-1), sucrose (Sucrose measurement kit A099-1-1), glucose (Glucose kit a154-1-1), and starch (Starch content kit A148-1-1) contents were measured using an assay kit (Nanjing Jiancheng Biotechnology Company, Nanjing, China). Units were expressed as mg metabolites per g of plant tissue. The experiment was conducted with three biological and three technical replicates.

### Measurement and Statistical Analysis of IAA, GA_3_, and BL Concentrations

Endogenous IAA, GA_3_, and BL were measured by liquid chromatography-mass spectrometry. Tetraploid and diploid stems and roots (leaves removed) were collected after 50 d of growth, then frozen in liquid nitrogen. IAA and GA_3_ detection methods were adopted from the work by Chen et al. (2012); the BL detection method was adopted from the work by Ding et al. (2013). Quantum triple quadrupole liquid chromatography-mass spectrometry (Thermo Fisher Scientific, Waltham, MA, United States) was used for plant hormone detection; three biological replicates were included in this experiment.

### Exogenous Hormone Addition

Adventitious shoots of tetraploid hybrid sweetgum were incubated in three types of new media for 0, 30, or 40 d. Each new medium contained one hormone: 1.5 mg/L IAA, 0.5 mg/L GA_3_, or 0.1 mg/L epibrassinolide. After determination of the optimal replacement time, the adventitious shoots were placed in rooting media that contained different hormones; eight combinations were used (Supplementary Table S2). There were three replicates in each treatment; each replicate contained 10 plantlets, including five genotypes. Each adventitious shoot was cut to a length of approximately 1.5 cm. After 60–70 d of growth (i.e., when the plant stopped growing), the plant height, ground diameter, internode number, main root number, and root length were measured.

### Statistical Analyses

Significant differences among treatments were evaluated by Duncan’s multiple range test using a threshold of *p* < 0.05, after a homogeneity test had been conducted; all statistical analyses were performed using SPSS version 18.0 (SPSS Inc., Chicago, IL, United States). Histograms and line graphs were constructed using Microsoft Excel 2010 software (Microsoft Corp., Redmond, WA, United States).

## Results

### Morphological Description of Regenerated Tetraploids and Diploids

**Figure 1:**
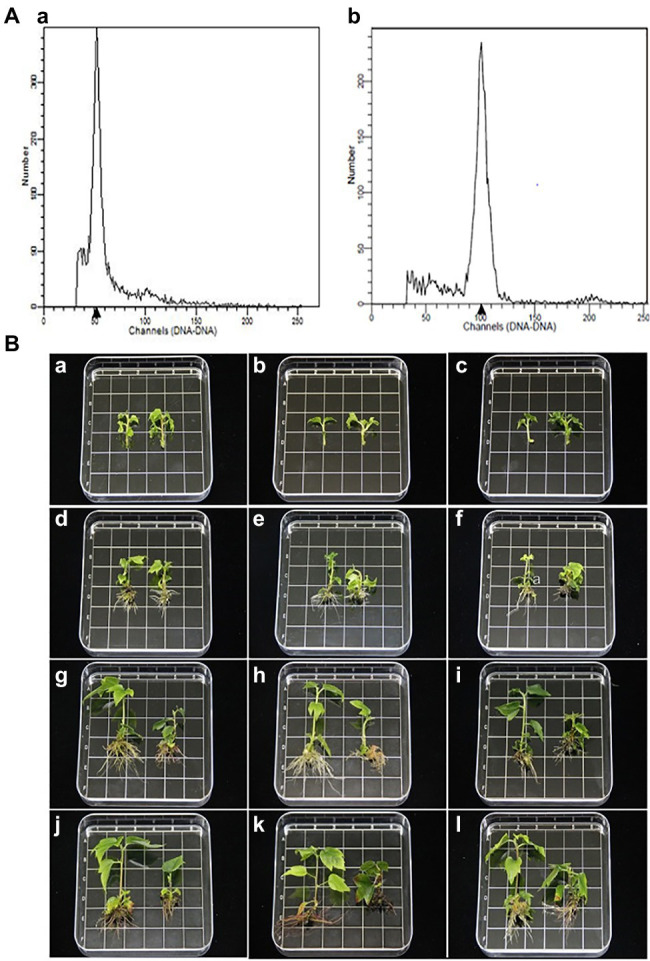
**(A)** Histograms of flow cytometry findings for *Liquidambar styraciflua* × *Liquidambar formosana*: **a** diploid plant (control); **b** tetraploid plant. **(B)** Morphological comparison of diploid and tetraploid hybrid sweetgum plants at various developmental stages of *in vitro* growth. **a,d,g,j** Z1; **b,e,h,k** Z2; **c,f,i,l** Z3. **a–c**: 0 d; **d–f**: 25 d; **g–i**: 50 d; **j–l** 70 d. **a–l** tetraploid on the right and diploid on the left in each picture. Area of each square: 1 × 1 = 1 cm^2^.

The ploidy levels of three genotypes were determined by flow cytometry before morphological and molecular examinations (Figure 1A). The phenotypes of tetraploid and diploid plants were measured after they had been cultured in rooting medium for 0, 25, 50, and 70 d (Table 1; Figure 1B). The results showed that there were no significant differences in ploidy among the plants.

**Table 1:** *In vitro* growth statistics of diploid and tetraploid hybrid sweetgum plants at various developmental stages.

	0		25 d		50 d		70 d	
	2X	4X	2X	4X	2X	4X	2X	4X
Plant height (mm)	15 ± 0c	15 ± 0c	19.35 ± 0.67c	17.5 ± 1.3c	36.28 ± 5.56b	19.14 ± 1.22c	41.05 ± 4.65a	20.07 ± 1.3c
Number of internodes	2.89 ± 0.19d	3.33 ± 0 cd	4.11 ± 0.69b	4 ± 0.33bc	5.44 ± 0.51a	3.89 ± 0.38bc	5.89 ± 0.38a	4.22 ± 0.19b
Stem diameter (mm)	1.24 ± 0.05c	1.47 ± 0.04bc	1.24 ± 0.12c	1.54 ± 0.09b	1.44 ± 0.3bc	1.48 ± 0.09bc	1.81 ± 0.07a	1.94 ± 0.17a
Root length (mm)	\	\	7.68 ± 1.72c	4.92 ± 0.66d	11.32 ± 1.62b	6.37 ± 1.03 cd	14.15 ± 0.81a	6.49 ± 0.8 cd
Root number	\	\	14.78 ± 2.5b	9 ± 0.33c	21.44 ± 2.22a	10.44 ± 1.35c	20.11 ± 2.59a	9.67 ± 0.33c
Number of leaves	4 ± 0.58f	4.44 ± 0.38ef	6.33 ± 0.58bc	5.44 ± 0.19d	6.78 ± 0.19ab	5.11 ± 0.38de	7.44 ± 0.51a	5.78 ± 0.19 cd
Leaf area (mm^2^)	18.74 ± 1.12c	23.67 ± 1.26c	19.99 ± 2.81c	28.54 ± 2.56c	63.32 ± 5.12b	57.5 ± 5.1b	87.05 ± 9.33a	84.25 ± 9.73a
Petiole length (mm)	2.09 ± 0.32bc	1.78 ± 0.02c	1.82 ± 0.11c	2.16 ± 0.3bc	2.49 ± 0.14b	2.25 ± 0.07bc	3.98 ± 0.33a	3.74 ± 0.6a

Data are represented as means ± standard errors of three replicates. Different lowercase letters indicate significant differences among treatments, as determined by Duncan’s test (*p* < 0.05).

In stage I, the height of tetraploids only increased by 1.93 mm, while the diploids grew rapidly; in stage II, a significant difference was apparent (Figure 1B). In stage III, the plant growth rates of both tetraploid and diploid plants were less than the rates in stage II (increases of 0.93 and 4.77 mm, respectively). At 50 d, the plant height and number of internodes were significantly shorter and fewer, respectively, in tetraploids than in diploids. In all three stages, the stem diameter was always larger in tetraploids than in diploids, but the difference was not statistically significant at 50 or 70 d. There were significantly fewer tetraploid leaves than diploid leaves at 25 d, and the area of tetraploid leaves was less than the area of diploid leaves at 50 d; however, there was no significant difference in petiole length at any developmental stage. The rooting times of tetraploid and diploid plants were similar; the root number and length were significantly fewer and shorter, respectively, in tetraploids than in diploids (Table 1; Figure 1B). These results showed that diploid growth was rapid in stage II, while tetraploid growth was not. Regenerated tetraploid plants were shorter and could grow fewer new buds after upper stem segments had been cut.

### Histological Observations of Leaves, Stems, and Roots of Regenerated Tetraploids and Diploids

**Figure 2:**
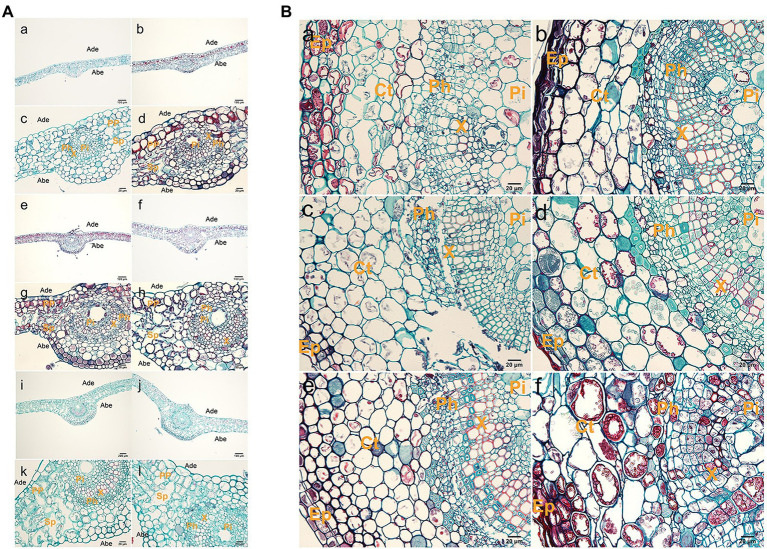
Histological observations of leaves and stems. **(A)** Anatomy of a leaf cross section from hybrid sweetgum. **a**,**c**,**e**,**g**,**i**,**k** Cross-section of a diploid leaf; **b**,**d**,**f**,**h**,**g**,**l** cross section of a tetraploid leaf. **a–d** 25 d; **e–h** 50 d; **i–l**: 70 d. Ade: adaxial epidermis; Abe: abaxial epidermis; PP: palisade parenchyma; Sp: spongy parenchyma; Pi: pith; Ph: phloem; X: xylem. Bars are 200 μm in **a,b,e,f,i,j**; they are 20 μm in **c,d,g,h,k,l**. **(B)** Anatomy of a stem cross section from hybrid sweetgum. **a**,**c**,**e** Cross section of a diploid shoot; **b**,**d**,**f** cross-section of a tetraploid shoot. **a**,**b** 25 d; **c**,**d** 50 d; **e,f** 70 d. Ct: cortex; Ep: epidermis; Pi: pith; Ph: phloem; X: xylem. Bars are 200 μm in **a,b,e,f**; they are 20 μm in **c**,**d**.

Observation of the second or third leaf positions of tetraploid and diploid plants showed that tetraploid leaves were significantly thicker than diploid leaves (Figure 2A; Supplementary Table S3). Tetraploid veins were also thicker than diploid veins, but this difference was not statistically significant at stage III. Palisade tissue and spongy tissue were also thicker in tetraploids than in diploids. The areas of xylem transverse cells, upper epidermal cells, lower epidermal cells, spongy tissue cells, and palisade tissue cells were larger in tetraploids than in diploids (Supplementary Table S4).

**Figure 3:**
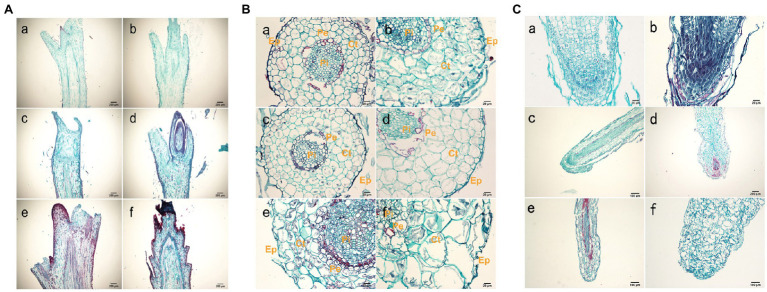
Histological observations of roots. **(A)** Anatomy of a shoot tip longitudinal-section from hybrid sweetgum. **a,** **c,** **e** Cross-section of a diploid shoot tip; **b,** **d,** **f** crosssection of a tetraploid shoot tip. **a,** **b** 25 d; **c,** **d** 50 d; **e,f** 70 d. Each bar is 200 μm. **(B)** Anatomy of a typical root cross section from hybrid sweetgum. **a,** **c,** **e** Cross-section of a diploid root; **b,** **d,** **f** cross-section of tetraploid roots. **a,** **b** 25 d; **c,** **d** 50 d; **e,** **f** 70 d. Ep: epidermis; Ct: cortex; Pi: pith; CC: central cylinder; Pe: pericycle. Bars are 100 μm in **a,b,e,f**; they are 20 μm in **c,** **d**. **(C)** Anatomy of a typical root tip longitudinal-section from hybrid sweetgum. **a,** **c,** **e** Cross-section of diploid root tips; **b,** **d,** **f** cross-section of tetraploid roof tips. **a,** **b** 25 d; **c,** **d** 50 d; **e,f** 70 d. Each bar is 200 μm.

The shoot tissue thickness and cell size were greater in tetraploids than in diploids. The tissue thickness and cell area increased with as the number of culture days increased (Figure 2B; Supplementary Tables S5 and S6). The shoot diameter, epidermal thickness, stem cortex thickness, and vascular column thickness were not significantly different between tetraploids and diploids, although epidermal thickness differed at 70 d. There were significant differences between tetraploids and diploids in terms of xylem cells at all three stages, cortex cells at 70 d, and phloem cells at both 50 and 70 d. Figure 2B shows that the apical meristem cells in diploid and tetraploid plants divided vigorously at 25 d; most diploid apical meristem cells continued to vigorously divide at 50 and 70 d (Figures 3Ac,e). However, most tetraploids exhibited a dormant bud apex at 50 d (Figure 3Ad). At 70 d, the apical uds were completely dormant and the bud scale structure was present (Figure 3A f). Therefore, the slow growth rate of the apical meristem may have contributed to shorter plant height in tetraploids than in diploids.

The root diameter and root tissue thickness at 25 and 50 d were larger in tetraploids than in diploids, with the exception of cylinder diameter at 70 d. The root diameter at 50 and 70 d, and the thicknesses of the epidermis and cortex at all three stages, significantly differed between tetraploids and diploids (Figure 3B; Supplementary Tables S7 and S8). The areas of xylem cells, epidermal cells, and cortical cells were significantly greater in tetraploids at all three stages. The apical meristematic cells of new diploid and tetraploid roots divided vigorously at 25 d of rooting culture (Figures 3Ca,b); the apical meristematic cells of diploid roots continued to vigorously divide at 50 d. However, most tetraploids exhibited increased root cell width and irregular shapes at 50 d (Figures 3Cc,d); the length of the root meristematic zone also decreased in tetraploids. The proportion of malformed roots was 81.33%. Complete coleoptile structure was not detected. These results indicated that root cell shape and root structure began to change after stage II in tetraploids; their longitudinal elongation ability significantly decreased.

### Transcriptome Sequencing and Assembly

Twelve samples were analysed in this study. The original quality score (Q30) of each sample was 93.45–95.07%, the effective data were 6.26–6.90 G, the mean guanine-cytosine (GC) content was 46.50%, and 6.81–7.48 G raw bases were obtained by sequencing (Supplementary Table S9). In total, 4 526,576 to 4 677,234 clean reads (Supplementary Table S9) were obtained after low-quality sequences had been filtered out. Furthermore, 60,187 Unigenes were assembled, with a total length of 5 200,279 bp and mean length of 917 bp (Supplementary Table S10). Of these sequences, 0.24–63.82% were annotated in seven databases: Nr, Swiss-Prot, KEGG, COG, evolutionary genealogy of genes: Non-supervised Orthologous Groups (eggNOG), GO, and Pfam (Supplementary Table S11).

### Analysis of DEGs

**Figure 4:**
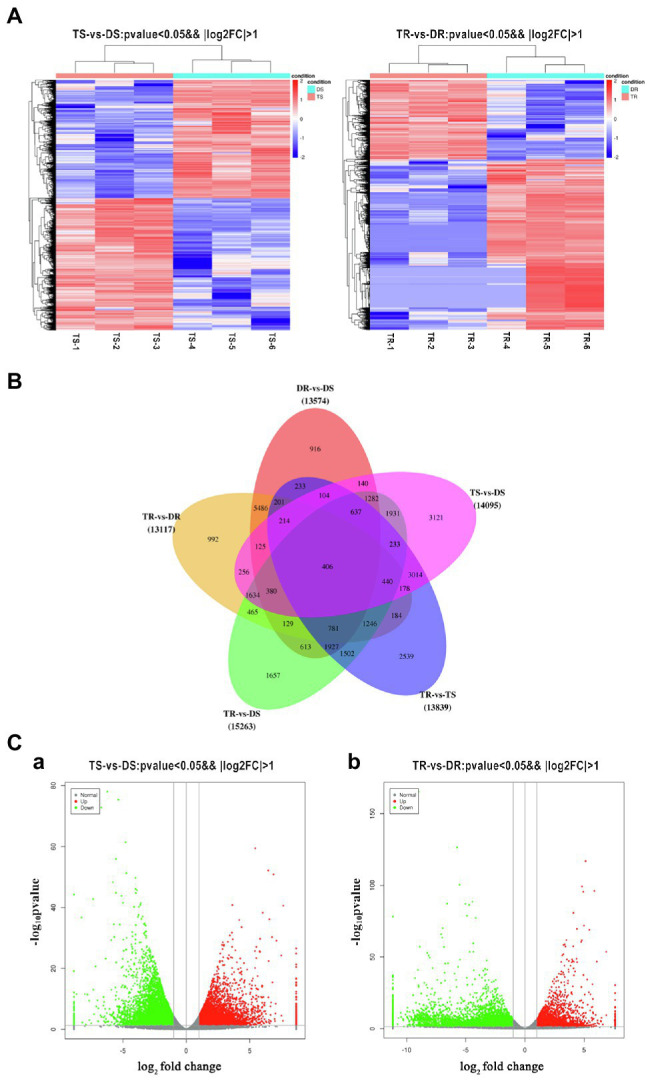
**(A)** Heatmap of DEG expression profiles (RNA-Seq) in tetraploid vs. diploid shoots. Left: DEGs were derived from TS-1_TS-2_ TS-3_vs DS-1_ DS-2_ DS-3. Right: DEGs were derived from TR-1_ TR-2_ TR-3_vs DR-1_ DR-2_ DR-3. Thresholds for DEG selection in the heatmap are *p* < 0.05 and |log_2_FoldChange| > 2. **(B)** Venn diagram analysis comparing five groups of DEGs. **(C)** Volcano plot of DEGs. **a,** **b** Represent the number of DEGs that were up- or downregulated for TS vs. DS and TR vs. DR, respectively. Grey indicates unigenes with similar expression patterns, red indicates significantly upregulated unigenes, and green indicates significantly downregulated unigenes. The x-axis is the log_2_FoldChange, and the y-axis is the log_10_*P*-value.

**Figure 5:**
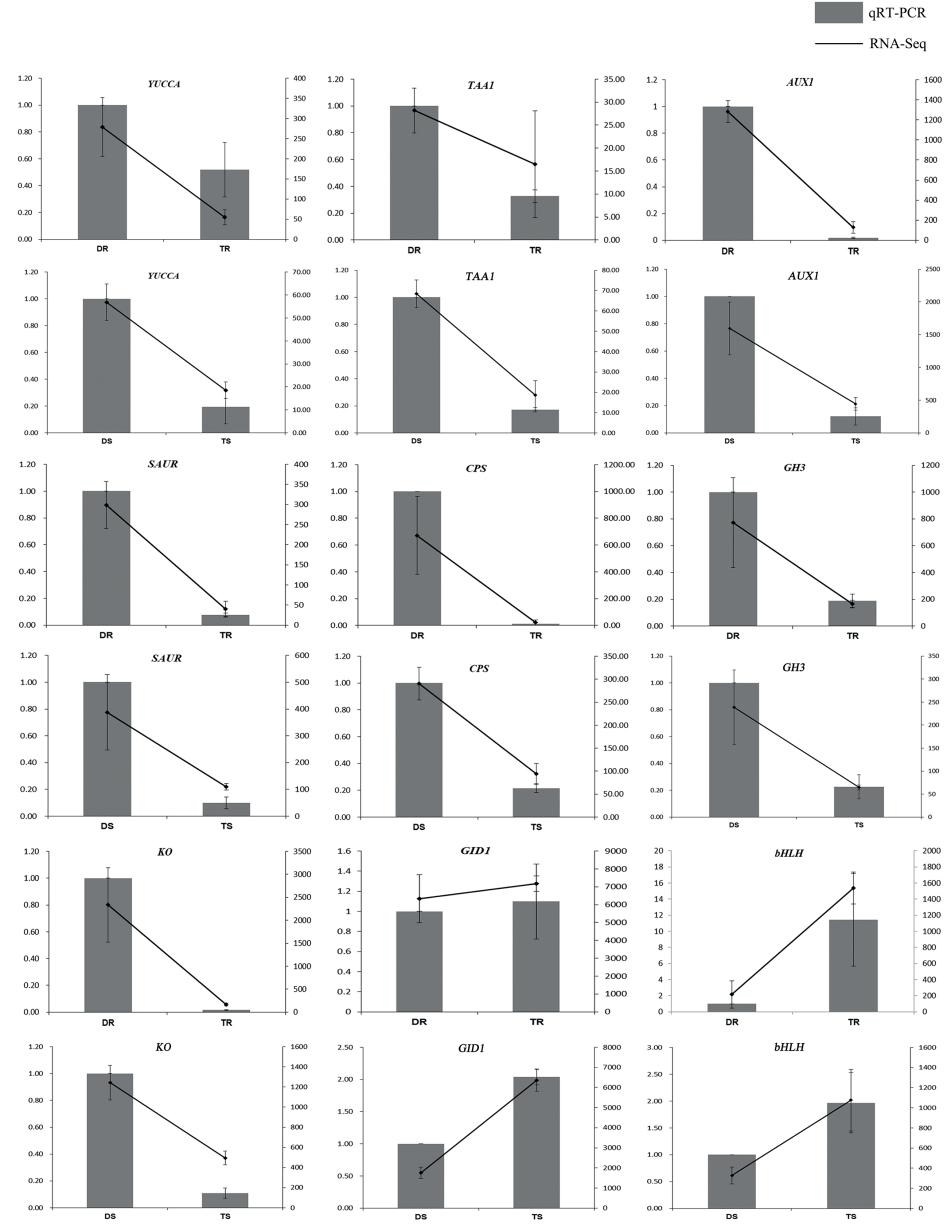
Comparison of expression profiles of nine representative genes as measured by RNA-seq and qRT-PCR. Columns represent expression determined by qRT-PCR (left y-axis), while lines represent expression by RNA-seq in FPKM values (right y-axis). The x-axis in each chart represents different plant materials. For qRT-PCR assay, the mean was calculated from three biological replicates each with three technical replicates (*n* = 9). For RNA-seq, each point is the mean of three biological replicates.

Statistical analysis of FPKM values in each group is shown in Supplementary Table S12 and Figure 4A. Overall, 14,095 and 13,117 DEGs were identified in tetraploid stems and roots, respectively, compared with diploid plants (Figure 4B). The qPCR results on selected genes were similar to the RNA-Seq data, which indicated that the results of RNA-Seq were reliable (Figure 5). In stems, 7,425 and 6,670 DEGs were upregulated and downregulated, respectively; in roots, 4,160 and 8,957 DEGs were upregulated and downregulated, respectively—these values constituted 31.71 and 68.29% of all DEGs, respectively (Figure 4C). Thus, gene expression was generally downregulated in the tetraploid roots of hybrid sweetgum.

### Analysis of DEG Enrichment in GO and KEGG Pathways

Gene ontology was used to analyse the DEGs of tetraploid and diploid stems and roots from three perspectives: biological process (BP), cell component (CC), and molecular function (MF). For stems and roots, DEGs were enriched in biosynthesis and signal transduction pathways, cell cycle, and carbon metabolism. For shoots, upregulated DEGs were enriched in guanosine triphosphate metabolic process (GO:0046039), gibberellin catabolic process (GO:0045487), other hormone biosynthesis and metabolic pathways, secondary metabolite biosynthetic process (GO:0044550), starch catabolic process (GO:0005983), and alkaloid metabolic process (GO:0009820; Supplementary Table S13). For shoots, downregulated DEGs were mainly enriched in histone phosphorylation (GO:0016572), cellular response to far red light (GO:0071490), cellular response to red light (GO:0071491), and histone kinase activity (H3-S10 specific; GO:0035175; Supplementary Table S14). For roots, upregulated DEGs were significantly enriched in maintenance of seed dormancy by abscisic acid (GO:0098755), response to low light intensity stimulus (GO:0009645), cellular response to freezing (GO:0071497), priming of cellular response to stress (GO:0080136), cellular response to boron-containing substance deprivation (GO:0080169), other stress response pathways, and NADH dehydrogenase complex (plastoquinone) assembly (GO:0010258; Supplementary Table S15). For roots, downregulated DEGs were mainly involved in septin ring assembly (GO:0000921) and cell cycle (GO:0007089), (GO:0071931, GO:2000045, GO:2000134; Supplementary Table S16).

### KEGG Analysis of DEGs

**Figure 6:**
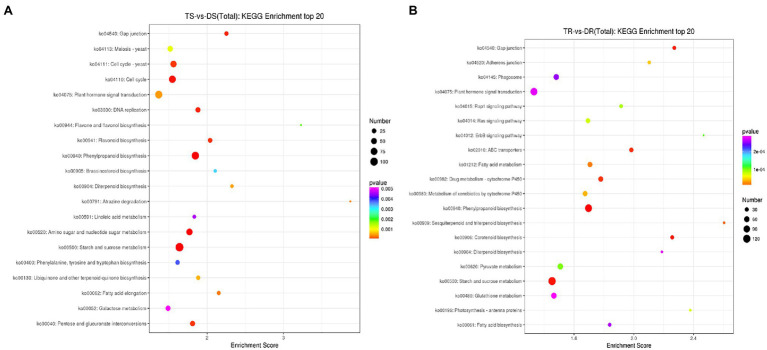
Map of KEGG enrichment analysis results for DEGs. **(A)** TS vs. DS; **(B)** TR vs. DR.

The KEGG database was used to identify biological pathways that were enriched in DEGs (Figure 6; Supplementary Tables S17 and S18). DEGs in tetraploid and diploid stems and roots were enriched in 47 and 72 biological pathways (*p* < 0.05), respectively. These pathways included gibberellin, auxin, brassinosteroids, salicylic acid, and ethylene pathways, such as plant hormone signal transduction (ko04075), diterpene biosynthesis (ko00904), brassinosteroids biosynthesis (ko00905) and carotenoid biosynthesis (ko00906); they also included secondary metabolite pathways, such as phenylpropionic acid synthesis (ko00940), flavonoid biosynthesis (ko00941), starch and sugar metabolism (ko00500), fatty acid synthesis and metabolism related to energy synthesis and metabolism (ko00071, ko01212, ko00061), cell cycle (ko04110), and apoptosis (ko04210).

### DEGs Related to Plant Hormones in Stems and Roots

**Figure 7:**
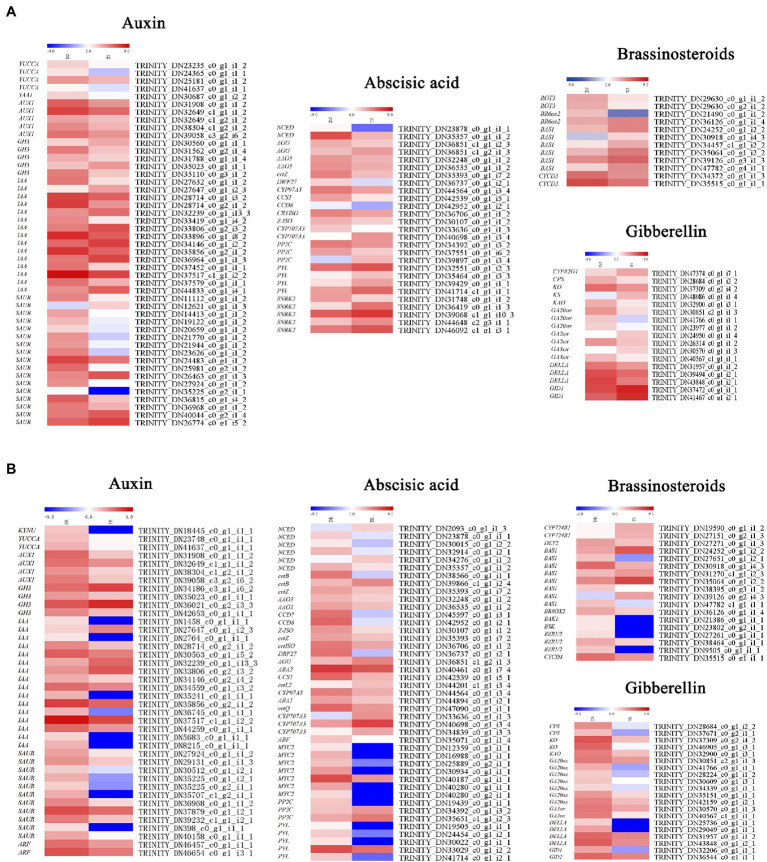
Heat maps depicting differential expression patterns of genes involved in plant hormone synthesis and signal transduction: **(A)** shoots, **(B)** roots.

Plant hormone biosynthesis and signal transduction pathways include auxin, gibberellin, cytokinin, BL, abscisic acid, ethylene, jasmonic acid and salicylic acid synthesis and transduction. Many important genes involved in auxin, gibberellin, and BL biosynthesis and signal transduction pathways were downregulated in tetraploids (Figure 7A). In shoots, the auxin biosynthesis genes *YUCCA* (*Indole-3-pyruvate monooxygenase YUCCA*) and *TAA1* (*Tryptophan aminotransferase of Arabidopsis1*) and the auxin influx carrier *AUX1* (*Auxin1*) were downregulated; *SAUR* (*Small auxin up RNA*), *IAA* (*Indole-3-acetic acid*), and *GH3* (*Gretchen hagan 3*) were generally downregulated (Figure 7A). Multiple gibberellin biosynthesis genes were also downregulated, including *CPS* (*Copalyl pyrophosphate synthase*), *KAO* (*Kaurenoic acid oxidase*), *KO* (*Kaurene oxidase*), *GA20ox* (*GA20-oxidase*), *GA2ox* (*GA2-oxidase*), and *GA3ox* (*GA3-oxidase*). The *GID1* (*GA-insensitive dwarf1*) gene, which contributes to positive regulation of gibberellin signal transduction, was upregulated; its negative regulation counterpart, *DELLA*, was generally downregulated. The BL biosynthesis and signal transduction genes *BAS1* (*phyB-4 activation-tagged suppressor 1*) and *CYCD3* (*Cyclin D3;1*) were downregulated. *ABA*, *PYL* (*Pyrabactin resistance 1-like*), the negative regulation *SnRKs* (*SNF1-related protein kinases*; TRINITY_DN36419_c0_g1_i1_3, TRINITY_ DN39068_c1_g1_i10_3, TRINITY_DN44648_c2_g3_i1_1, TRINITY_ DN46092 _ c1 _g1_i3_1)—which negatively regulates abscisic acid signal transduction—and *PP2C* (*Protein phosphatase 2C*; TRINITY_DN34392_c0_g1_i3_2 and TRINITY_DN37551_c0_g1 _i6_2) were downregulated. However, the positive regulation counterpart, *ABF* (*Abscisic acid responsive elements-binding factor*), was generally downregulated.

Similar to stems, the auxin biosynthesis genes *YUCCA* and *KYNU* (*kynureninase*), auxin signal transduction genes *AUX/IAA* (auxin/indole-3-acetic acid), *SAUR*, *GH3* (TRINITY_DN35023 _c0_g1_i1_1, TRINITY_DN42653_c0_g1_i1_1) and *IAA* were downregulated in roots, while auxin influx carrier *AUX1* was also similarly downregulated. The gibberellin biosynthesis biosynthetic genes (e.g., *CPS*, *KO*, *KAO*, *GA20ox*, and *GA3ox*) were all downregulated. However, *GID1*, which positively regulates gibberellin signal transduction, was upregulated; *DELLA*, its negative regulation counterpart, was downregulated. For ABA, except CYP707A3, the biosynthesis genes *AAO3* and *ABA2* were downregulated, while *NCED* (*9-cis-epoxycarotenoid dioxygenase*) was generally downregulated. In contrast to stems, with the exception of *PP2C*, the signal transduction genes *PYL*, *SnRKs*, and *ABF* were generally downregulated (Figure 7B).

### DEGs Related to Starch and Sucrose Biosynthesis and Metabolism

**Figure 8:**
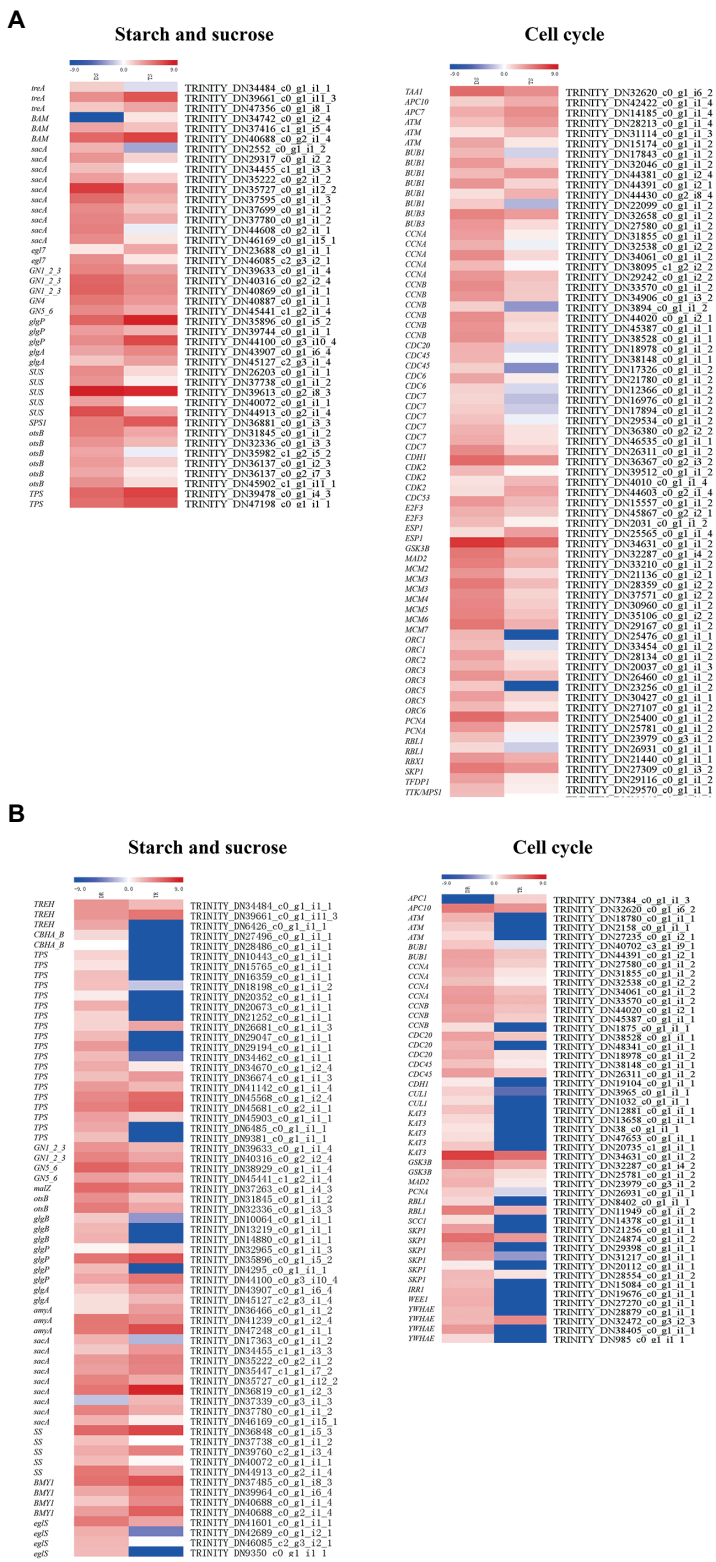
Heat maps depicting DEGs involved in starch and sucrose biosynthesis and metabolism and cell cycles: **(A)** shoots, **(B)** roots.

In stems and roots, there were 42 and 65 DEGs related to starch and sugar metabolism, respectively (Figure 8); most were downregulated. In stems, sacA, *SS* (*Starch synthase*), glgA, otsB, GN1_2_3, GN4, GN5_6, SPS1 (*Sucrose-phosphate synthase1*), and *SUS* (*Sucrose synthase*) were downregulated; TPS (*Trehalose 6-phosphate synthase*) was upregulated. In roots, *TPS*, *SS* (TRINITY_DN 37738_c0_g1_i1_2, TRINITY_DN40072_c0_g1_i1_1, TRINITY_DN44913_c0_g2 _i1 _4), *sac* (TRINITY _DN 17363 _c 0_g1_i1_2, TRINITY_DN35727_c0_g1_i12_2, TRINITY_DN36819_c0_g1_i2_3, TRINITY_DN46169_c0_g1_i15_1), *otsB*, *GN1_2 _3*, and *GN5_6* were downregulated.

### DEGs Related to Cell Cycles in Stems and Roots

There were 76 and 49 genes involved in regulating cell cycles (ko04110) in stems and roots, respectively (Figure 8). In stems, these genes were generally downregulated, except *ATM* (*Ataxia telangiectasia mutated*), *CDK2* (*cyclin-dependent kinase 2*), *CDC6* (cell division control protein 6), *CDC7*, *CDC20*, *CDC45*, *E2F3*, *Bub1*, *Bub3*, *CycA* (*Cyclin A*), *Orc1-Orc3*, and *Orc5*; notably, *Orc6*—a contributor to cell proliferation and cell cycle regulation—was generally downregulated. In roots, except for two genes, the DEGs were downregulated in tetraploids, including *ATM*, *BUB1*, *CDC20*, *CDC45*, *CUL1* (*Cullin 1*), *SKP1* (*S-phase kinase-associated protein 1*), and *GSK3B* (*glycogen synthase kinase 3 beta*). These genes have important roles in regulating cell size and cell number. Therefore, the generally larger cells of tetraploid hybrid sweetgum, compared to diploid hybrid sweetgum, may be related to significant changes in cell cycle genes.

### Analysis of the Hormone and Sugar Contents of Tetraploids and Diploids

**Figure 9:**
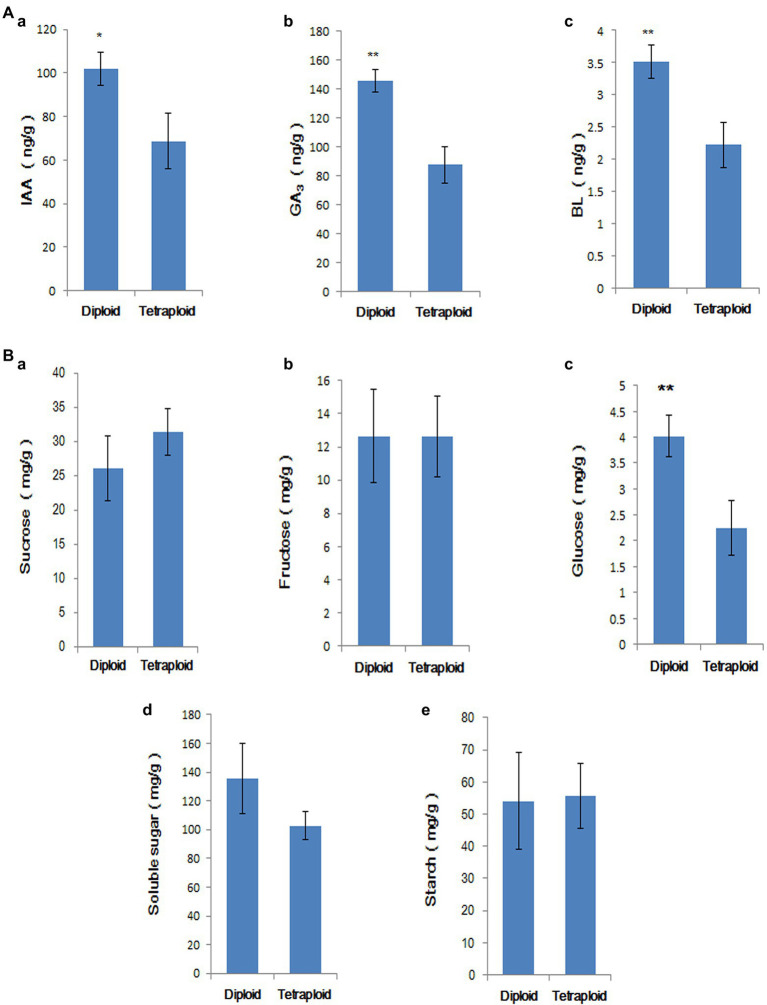
**(A)** Comparison of IAA (a), GA_3_ (b), and BR (c) contents in tetraploid and diploid stems and roots. ‘*’ indicates *p* < 0.05, ‘**’ indicates *p* < 0.01. **(B)** Comparison of sucrose (a), fructose (b), glucose (c), soluble sugar (d), and starch contents (e) in tetraploid and diploid stems and roots. ‘**’ indicates *p* < 0.01.

The IAA, GA_3_, and BL contents of diploid and tetraploid roots and stems cultured in rooting medium for 50 d were measured; the results are shown in Figure 9A. The endogenous auxin, gibberellin, and BL levels were significantly lower in tetraploids than in diploids, consistent with the results of the transcriptome analysis. Compared with diploids, only the glucose contents significantly differed in tetraploids (Figure 9B). The result showed that although starch and sugar biosynthesis genes were downregulated in tetraploid plants, the starch and soluble sugar contents did not significantly decrease. Therefore, gene expression and sugar accumulation were not synchronised at this stage.

### Effects of Exogenous Hormones on Plant Growth

To verify the effects of IAA, GA_3_, and BL on elongation in tetraploids, the effect of the timing of tetraploid transplantation into new medium was investigated. As shown in Supplementary Table S19, the growth of regenerated tetraploid plants was only significantly promoted at the initial stage when the rooting medium was supplemented with 0.5 mg/L GA_3_ or 1.5 mg/L IAA. These results showed that the timing of addition of exogenous hormones significantly affected the growth of regenerated plants. As time elapsed without replacement of the medium, the elongation effect gradually decreased.

**Figure 10:**
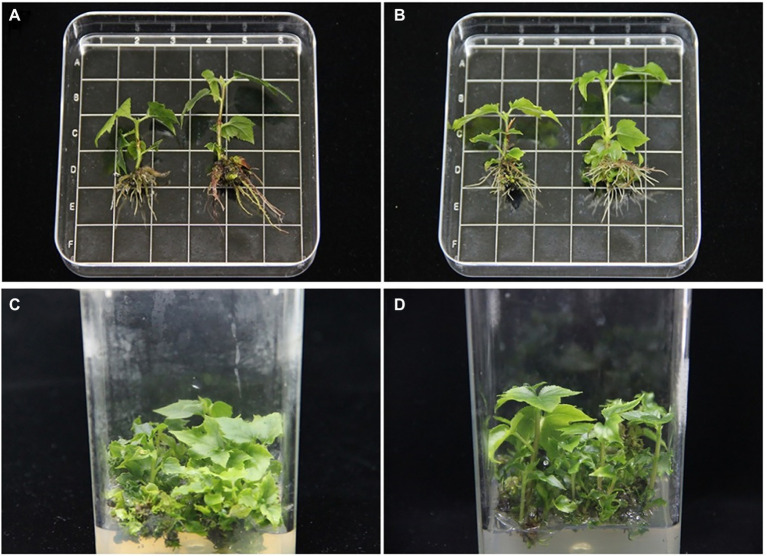
Effects of GA_3_ and IAA on shoot and root lengths of tetraploid hybrid sweetgum Z4 grown *in vitro* **(A,B)**. **(A)** Control on the left (primitive rooting medium) and rooting medium supplemented with 0.5 mg/L GA_3_ on the right. **(B)** Control on the left (primitive rooting medium) and rooting medium supplemented with 0.5 mg/L GA_3_ and IAA on the right. Area of each square: 1 × 1 = 1 cm^2^. Effects of GA_3_ on adventitious bud length in tetraploid hybrid sweetgum **(C,D)**. **(C)** Control. **(D)** Development of adventitious shoots in medium supplemented with 0.5 mg/L GA_3_.

After determination of the optimal medium replacement time, the effects of media containing different hormone ratios on tetraploid growth were investigated. The results showed that the greatest plant height was 4.42 cm in a rooting medium supplemented with 0.5 mg/L GA_3_; the plant height, ground diameter, root length, and leaf area were significantly increased compared with tetraploid hybrid sweetgum grown in primitive rooting medium, but the number of roots did not significantly differ (Figure 10; Supplementary Table S20). The plants placed in a medium with the addition of 0.5 mg/L GA_3_ combined with 1.5 mg/L IAA had the largest leaf area and greatest number of main roots. Therefore, GA_3_ was the hormone that most strongly supported stem elongation (Supplementary Table S20). The effects of BR alone or in combination with GA_3_ or IAA were weaker than the effects of other two hormones. These results indicated that the height of tetraploid plants could be significantly increased by adding an appropriate concentration of GA_3_ to the growth media. In addition, the stem segments used for subcultures could be rooted and elongated normally in the medium. Therefore, *in vitro* micro-cutting could be used to subculture and expand tetraploid plants. Additionally, 0.5 mg/L GA_3_ was added to the tetraploid adventitious bud elongation medium; it demonstrated a strong effect on adventitious bud elongation (Figure 10).

## Discussion

Tetraploid hybrid sweetgum regenerated plants exhibited obvious morphological variations, with shorter stems and roots than diploid plants. Furthermore, the stem diameter and internode length were thicker and shorter, respectively, in tetraploids than in diploids. These observations were consistent with previous findings in some herbs, fruit trees, and timber trees (Nilanthi et al., 2009; Tan et al., 2015; Xu et al., 2018). However, with the exceptions of stems and roots, the other organs of dwarf tetraploids did not necessarily exhibit a growth disadvantage. The plant height was shorter in tetraploid apples than in diploid apples, but the leaf and flower organs were obviously larger in tetraploid apples (Xue et al., 2017). Thus, plant height is not necessarily positively related to the sizes of other organs. In addition, Corneillie et al. (2019) found that in *Arabidopsis thaliana*, the leaf area, stem length, and dry weight were greater in tetraploid plants than in plants with other ploidy levels; as the ploidy level increased, hexaploid and octoploid plants gradually exhibited less growth. The ploidy level at which optimal growth is reached differs among species.

The proliferation and enlargement rates of apical and intersegment meristematic cells have considerable effects on elongation and development in roots and stems (Takatsuka and Umeda, 2014). The relationships among organ size, cell size, and cell number remain unresolved (Tsukaya, 2008; Li et al., 2012). Previous studies have suggested that plant organ sizes are determined by both cell size and cell number (Krizek, 2009; Gonzalez et al., 2012). Generally, plant cell sizes significantly increase after chromosome doubling. Endoreduplication is considered an important cause of polyploid cell enlargement (Tsukaya, 2008). The numbers of cells vary among species and ploidy levels. Compared with diploids, triploid *Populus* plants showed a significant increase in cell area, but the number of cells did not decrease (Zhang et al., 2019). A similar observation was reported in a study of *A. thaliana* (Li et al., 2012). However, cell area was larger in octoploid *Arabidopsis* plants than in diploid plants, but the smaller number of cells resulted in a smaller leaf area (Levan, 1939). These results indicated that the cell size was greater in tetraploids than in diploids, but the tetraploid plants had dwarfing characteristics.

In the present study, tetraploid hybrid sweetgum root cells exhibited increased width and irregular shape, the tetraploid root meristematic zone length decreased, and no obvious coleoptile sheath or complete coleoptile structure were observed. Few observations of root malformation have been reported in tetraploid regenerated plants; however, in apples, the ratio of length to width of parenchyma cells is altered in the stem cortex (Ma et al., 2016). Compared with diploids, the apical bud entered dormancy earlier; it was difficult for most plants to exit dormancy and continue to elongate, which may explain why tetraploid regenerated plants were shorter than diploid plants. Sun et al. (2016) found that only a few individuals had autumn shoot growth, but none of these individuals were diploid. Changes in the content and proportion of endogenous hormones in plants are closely related to bud dormancy (Tanino, 2004; Ruttink et al., 2007), especially the ratio of gibberellin to abscisic acid (Rinne et al., 2011). In this study, the morphological variations of roots and stems may be closely related to hormone levels in tetraploid hybrid sweetgum; changes in the hormone ratio may lead to premature dormancy in tetraploid regenerated plants.

After polyploidization, growth conditions usually change *in vitro*; thus, the original medium for diploids is often unsuitable for tetraploid plant growth. For example, *Lagerstroemia crassipes* is difficult to root in an original culture medium (Tong, 2007). Root and stem elongation is an important process in plant tissue culture, but the elongation of adventitious shoots and plantlets is problematic in many species. Plant hormones (e.g., auxin, gibberellin, and BL) are the most important factors that affect the lengths of regenerated organs; such hormones can promote *in vitro* plant elongation (Sasaki, 2002; Zhang et al., 2013, 2018). The concentrations of auxin hormones (e.g., IAA and indole butyric acid) added to the medium have a significant effect on root length elongation (Nandagopal and Kumari, 2007). In this study, the rooting medium supplemented with IAA alone promoted roots of tetraploid hybrid sweetgum elongation; it also increased plant height. This result may have occurred because, although the polar transport of auxin occurs from top to bottom, the growth processes of underground and aboveground parts often interact. An increase in underground growth can provide more water, inorganic salts, organic substances, and hormones for the shoots (Agren and Franklin, 2003). In this study, the number of roots was significantly fewer in tetraploids than in diploids; a similar phenomenon has been reported, but in *Gerbera jamesonii*, the plant height was significantly greater in tetraploids than in diploids (Gantait et al., 2011). Therefore, tetraploid plant height may not be directly proportional to root number.

In the present study, gibberellin had effects on root and stem of tetraploid hybrid sweetgum elongation; however, the induction of root thinning by gibberellin may have been related to the gradient change in roots. Eiichi (2012) suggested that the gibberellin-mediated-concentration dependent-stimulation of elongation is important for regulating plant height and root length; GA_3_ could also significantly promote elongation in adventitious buds of sweetgum (Wang et al., 2018b).

Brassinolide also has important roles in promoting cell elongation, cell enlargement, and vascular tissue differentiation (Clouse, 2002). Previous studies have shown that BL can promote both lateral stem expansion and vertical stem growth (Sasse, 1997). Clouse and Sasse (1998) found that a low concentration of BL helped to accelerate cell division, whereas high concentrations had the opposite effect. In the present study, the addition of 0.1 mg/L BL did not have a significant effect on the lengths of roots and stems. Therefore, the concentration range is presumably unsuitable for tetraploid hybrid sweetgum elongation.

Previous studies have shown that reduced expression levels of genes with positive regulatory roles in gibberellin, auxin, and BL biosynthesis and signal transduction are important causes of plant dwarfing (Li et al., 2017; Ju et al., 2018; Wang et al., 2018a). Ma et al. (2016) suggested that changes in the auxin and BL contents in tetraploid apples might contribute to plant height variation. In the present study, the DEGs of tetraploid and diploid hybrid sweetgum stems and roots were also highly enriched in the hormone synthesis and signal transduction pathways, which were related to auxin, gibberellin, and BL levels.

Although the auxin synthesis pathway has not been fully analysed, an in-depth analysis of the indole-3-pyruvate pathway has been conducted. In the present study, the significant downregulation of *YUCCA* and *TAA1* genes of tetraploid hybrid sweetgum had important effects on auxin biosynthesis. TAA participates in the transformation of tryptophan (Trp) into indole-3-pyruvate, while YUCs participate in the transformation of indole-3-pyruvate into IAA (Tao et al., 2008; Won et al., 2011; Zhao, 2012). *YUC* and *TAA* genes affect the growth of the *yuc* and *tar* mutants of *A. thaliana*, which have very short hypocotyls and roots (Won et al., 2011). Auxin signal transduction genes are classified into *AUX*/*IAA*, *SAUR*, and *GH3* groups (Guilfoyle and Hagen, 2007), which have important roles in plant growth and development. *AUX/IAA* is an early auxin response gene, and its protein product can specifically bind to auxin response factor (ARF), thereby regulating the expression of auxin response genes; in the *A. thaliana* mutant *AUX*/*IAA*, the lengths of the stem and hypocotyl are altered; apical dominance and root formation are also modified (Liscum and Reed, 2002). In the present study, auxin signal transduction genes (e.g., *AUX*/*IAA*, *GH3*, and *SAUR*) and auxin influx carrier *AUX1* (Swarup and Bhosale, 2019) were substantially downregulated in roots and stems.

Gibberellin biosynthesis genes also affect cell division and elongation, thereby influencing plant stem and root elongation. In this study, the *CPS*, *KAO*, *KO*, *GA20ox*, *GA2ox*, and *GA3ox* genes were generally downregulated in tetraploid hybrid sweetgum. These genes are involved in the three main steps of gibberellin synthesis. First, ent-kobaki pyrophosphate synthase (CPS) and ent-kaurene synthase catalyse the formation of ent-kaurene from yak’s geranyl pyrophosphate. Second, ent-kaurene catalyses the formation of GA12 and GA53 by ent-kaurene oxidase (KO) and ent-kaurenoic acid oxidase (KAO). Third, the oxidases GA20ox, GA2ox, and GA3ox eventually catalyse GA12 and GA53 to form GA1 and GA4 (Hedden and Phillips, 2000). Gibberellin and auxin are closely related and have significant interaction (Depuydt and Hardtke, 2011; Willige et al., 2011). Auxin reportedly can promote root growth in *A. thaliana* by acting on the gibberellin responsive protein, DELLA (Fu and Harberd, 2003). *GID1* and *DELLA* have key roles in gibberellin signal transduction. After activation by gibberellin, the gibberellin receptor GID1 binds to DELLA *via* the E3 ubiquitination pathway, which leads to degradation of the DELLA protein and thus eliminates growth inhibition (Sun, 2010). However, in maize, *DELLA* deletion mutants showed dwarfing traits (Lawit et al., 2010). Therefore, although DELLA is a negative regulator, it is indispensable.

Changes in the expression patterns of BL biosynthesis and signal transduction genes also have significant effects on plant height and root length. *DET2*, *ROT3*, and *BR6ox1* are important BL biosynthesis genes (Noguchi et al., 2000; He et al., 2005). A balanced BR signal is necessary to maintain the root meristem (Takatsuka and Umeda, 2014). The root meristem length of the *bri1-116* mutant is decreased, but overexpression of the cell cycle gene *CYCD3*;*1* inhibits this decrease (Gonzalez-Garcia et al., 2011). The overexpression of the mutant *BZR1* significantly promoted hypocotyl elongation and increased plant height (Wang et al., 2002).

Previous studies have shown that epigenetic changes are important sources of transcriptional variation after polyploidization (Ni et al., 2009; Chen, 2013). The downregulation of the auxin signal transduction gene *MdARF3* in tetraploid apples may be related to upregulation of the negative regulator *MiR390*; it may affect auxin signal transduction (Ma et al., 2016). Therefore, significant changes in the expression levels of hormone-related genes in tetraploid hybrid sweetgum may be related to the upregulation or downregulation of small RNA genes.

**Figure 11:**
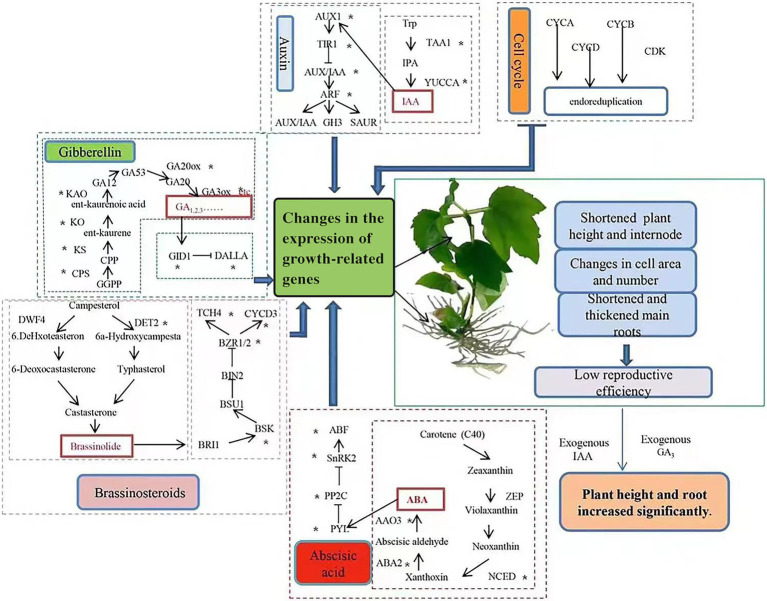
Effects of changes in endogenous hormone biosynthesis and signal transduction genes and supplementation with exogenous hormones on tetraploid hybrid sweetgum growth. ‘*’ indicates DEG enrichment in roots or stems.

Based on our findings, we constructed a schematic of the effects of changes in endogenous hormone biosynthesis and signal transduction genes, as well as supplementation with exogenous hormones, on growth in tetraploid hybrid sweetgum (Figure 11). As discussed previously, reduced expression levels of genes in gibberellin, auxin, BL and other hormone biosynthesis and signal transduction are important causes of tetraploid hybrid sweetgum dwarfing. Furthermore, the down-regulated expression of important genes involved in cell cycle may also have an impact on tetraploid hybrid sweetgum. Exogenous GA_3_ and IAA, which induced plant cell elongation, could significantly promote growth in the stems and roots of tetraploids. It is currently unclear why some species exhibit two different phenotypes (i.e., fast growth or slow growth) after polyploidization. Species evolution and genome duplication induce gene redundancy. Epigenetic modification models differ among species; such models may influence the expression patterns of genes related to growth and development. This complex problem should be investigated in future studies.

## Conclusion

To our knowledge, this is the first investigation of the polyploid molecular mechanism in the dwarf type of hybrid sweetgum grown *in vitro*. Genes that promote organ growth (e.g., auxin, gibberellin, and BL biosynthesis) and genes involved in signal transduction were downregulated. These changes may be the main cause of root and stem variations in tetraploids, which lead to slower growth in tetraploids than in diploids. Both IAA and GA_3_ can significantly promote shoot and root elongation in dwarf tetraploid hybrid sweetgum. Our findings provide insights into the molecular mechanisms that underlie dwarfism in allopolyploid hybrid sweetgum.

## Data Availability Statement

The data presented in the study are deposited in the NCBI repository, accession number PRJNA831658.

## Author Contributions

JZhan and JZhao designed the study. YZ and SC conducted the experiments, analysed the data, and drafted the manuscript. SC, YZ, TZ, JZhao, and ZP critically reviewed and improved the manuscript. All authors contributed to the article and approved the submitted version.

## Funding

This work was supported by National Forestry and Grassland Administration Promotion Project of China (2020133102); Scale-up Propagation Technology Promotion *via* Somatic Embryogenesis of Sweetgum Forestry Science and Technology Innovation Special Project of Jiangxi Province (2019-16); the Fundamental Research Funds for the Central Universities (2019ZY39); Major Science and Technology Special Project of Xuchang, Henan province, China (20170112006).

## Conflict of Interest

DZ and ZP are employed by Guangxi Bagui Forest and Flowers Seedlings Co., Ltd.

The remaining authors declare that the research was conducted in the absence of any commercial or financial relationships that could be construed as a potential conflict of interest.

## Publisher’s Note

All claims expressed in this article are solely those of the authors and do not necessarily represent those of their affiliated organizations, or those of the publisher, the editors and the reviewers. Any product that may be evaluated in this article, or claim that may be made by its manufacturer, is not guaranteed or endorsed by the publisher.

## Supplementary Material

The Supplementary Material for this article can be found online at: https://www.frontiersin.org/articles/10.3389/fpls.2022.924044/full#supplementary-material

## Footnotes

^1^ http://rsb.info.nih.gov/ij/

^2^ http://www.geneontology.org/

^3^ http://www.genome.jp/kegg/kaas/
