# Supplementary material for: Comparative Transcriptomic, Anatomical and Phytohormone Analyses Provide New Insights Into Hormone-Mediated Tetraploid Dwarfing in Hybrid Sweetgum (Liquidambar styraciflua × L. formosana)
Source: Front Plant Sci. 2022 Jun 27;13:924044. doi: 10.3389/fpls.2022.924044 (PMC9271929; doi:10.3389/fpls.2022.924044)
Supplement: Supplementary file 1 [file Table_1.DOCX]

Supplementary Material

**Table S1** Primer sequence of qRT-PCR information

| **No.** | **Gene Symbol** | **Gene name** | | **Forward primer** | **Reverse primer** | **Amplified fragment length (bp)** |
| --- | --- | --- | --- | --- | --- | --- |
| 1 | DN41637  _c0_g1_i1_1 | *YUCCA* | CTCCCAAAGC  AATTCTGTGA | | ATCCTCCAAG  TATCCAATGAAC | 96 |
| 2 | DN30687  _c0_g1_i2_2 | *TAA1* | CTGTATAAAT  GGGCTGGTGATG | | GGGTGAAGTT  ACAAGCTCAA | 72 |
| 3 | DN31908  _c0_g1_i1_2 | *AUX1* | CCATCAGCTT  CTGCTGTTTAT | | ATGAATGAGC  ATGAGGACG | 120 |
| 4 | DN36968  _c0_g1_i1_2 | *SAUR* | TCATGTTGGG  TGCTTTAGAGT | | GACCTATTCT  TGGTTATGCCAG | 70 |
| 5 | DN28684  _c0_g1_i2_2 | *CPS* | ACCTTGCTCG  GAACTCTAA | | TTTCCCAAGC  GTGACGTA | 91 |
| 6 | DN32900  _c0_g1_i3_1 | *GH3* | GGAGTTGGGT  TTCGCTTC | | TGCTCAGCAC  CCACTGTA | 74 |
| 7 | DN37309  _c0_g2_i4_2 | *KO* | GGCTGGTTGT  CCAAACTT | | CACCTTGTGAA  CGTCTTGTAT | 107 |
| 8 | DN41467  _c0_g1_i2_1 | *GID1* | AATTATCGCC  GATCACCTG | | TTAAGAGCTG  TCCATCCATCAT | 65 |
| 9 | DN37065  _c0_g1_i1_1 | *bHLH* | CCGAGTCGAG  ATTTGCTG | | GGCCTAATGC  TTCTAGGGTA | 74 |
| 10 | Internal reference gene | 18S | TGCTTTCGCAGT  TGTTCGTCTTTCA | | GCAAGCCTACGC  TCTGGATACATTA | 405 |

**Table S2** Combination of rooting medium with different hormones

|  | **IAA（mg/L）** | **GA（mg/L）** | **EBR（mg/L）** |  |
| --- | --- | --- | --- | --- |
| 1 | 0 | 0 | 0 | |
| 2 | 1.5 | 0 | 0 | |
| 3 | 0 | 0.5 | 0 | |
| 4 | 0 | 0 | 0.1 | |
| 5 | 1.5 | 0.5 | 0 | |
| 6 | 1.5 | 0 | 0.1 | |
| 7 | 0 | 0.5 | 0.1 | |
| 8 | 1.5 | 0.5 | 0.1 | |

**Table S3** Comparison of leaf anatomy between diploid and tetraploid of hybrid sweetgum

| Thickness (μm) | **days** | **2x** | **4x** |
| --- | --- | --- | --- |
| Blade | 25 d | 105.86±3.44 cd | 145.72±14.09 ab |
|  | 50 d | 97.62±8.03 d | 143.51±16.66 ab |
|  | 70 d | 122.99±14.79 bc | 150.94±11.85 a |
| Main vein | 25 d | 200.32±21.15 d | 271.28±29.73 bc |
|  | 50 d | 246.46±11.27 cd | 278.49±40.98 bc |
|  | 70 d | 316.61±21.28 ab | 355.94±50.67 a |
| Palisade tissue | 25 d | 20.94±1.9 c | 33.18±2.61 ab |
|  | 50 d | 19.6±0.83 c | 30.29±2.13 b |
|  | 70 d | 32.32±5.75 ab | 38.67±4.85 a |
| Spongy tissue | 25 d | 73.99±6.27 ab | 93±9.44 a |
|  | 50 d | 62.96±10.02 a | 86.49±14.44 ab |
|  | 70 d | 80.1±9.91 abc | 96.61±4.48 a |

Note: Data are represented as the mean ± SE of three replicates. Different lowcase letters indicate significant differences among treatments as determined by Duncan’s test（*p* ≤ 0.05）.

**Table S4** Comparison of leaf cell characteristics between diploid and tetraploid hybrid sweetgum

| **Cell Area** (μm^2^) | **d** | **2x** | | **4x** |
| --- | --- | --- | --- | --- |
| Xylem cell | 25 d | 49.86±18.92 c | | 85.59±18.25 ab |
|  | 50 d | 56.11±7.82 bc | | 92.31±12.85 a |
|  | 70 d | 83.63±15.88 ab | | 76.54±20.97 abc |
| Upper epidermal cells | 25 d | | 207.58±43.36c | 372.19±67.57ab |
|  | 50 d | | 268.64±73.44bc | 411.24±63.27ab |
|  | 70 d | | 273.15±15.97bc | 475.66±157.67a |
| Lower epidermal cells | 25 d | | 109.99±30.40 c | 168±34.71ab |
|  | 50 d | | 135.23±30.66bc | 176.56±26.08ab |
|  | 70 d | | 160.66±27.56bc | 217.5±21.56a |
| Spongy tissue | 25 d | | 182.33±60.5c | 318.72±51.69a |
|  | 50 d | | 173.48±40.08c | 304.93±24.61ab |
|  | 70 d | | 209.04±105.69bc | 335.06±23.84a |
| Palisade tissue | 25 d | | 178.41±54.92c | 437.91±57.45a |
|  | 50 d | | 170.01±31.57c | 457.23±41.89a |
|  | 70 d | | 260.45±11.24b | 481.92±20.57a |

Note: Data are represented as the mean ± SE of three replicates. Different lowcase letters indicate significant differences among treatments as determined by Duncan’s test（*p* ≤ 0.05）.

**Table S5** Comparison of stem anatomy between diploid and tetraploid of hybrid sweetgum

| Thickness (μm) | **d** | **2x** | **4x** |
| --- | --- | --- | --- |
| Diameter | 25 d | 1069.93±96.72b | 1234.33±73.43b |
|  | 50 d | 1113.72±109.55b | 1403.39±350.3ab |
|  | 70 d | 1339.9±140.18ab | 1681.7±211.57a |
| Epidermis | 25 d | 19.49±2.36b | 22.85±2.43b |
|  | 50 d | 21.99±2.11b | 21.61±8.12b |
|  | 70 d | 22.23±4.67b | 31.86±3.84a |
| Cortex | 25 d | 181.74±6.52b | 210.68±11.66ab |
|  | 50 d | 177.84±26.44b | 216±37.94ab |
|  | 70 d | 205.47±53.87ab | 261.85±66.84a |
| Cylinder | 25 d | 612.13±28.15b | 741.95±52.65b |
|  | 50 d | 740.02±15.26b | 897.53±265.03ab |
|  | 70 d | 848.59±16.79ab | 1145.8±332.29a |

Note: Data are represented as the mean ± SE of three replicates. Different lowcase letters indicate significant differences among treatments as determined by Duncan’s test（*p* ≤ 0.05）.

**Table S6** Comparison of stem cell characteristics between diploid and tetraploid hybrid sweetgum

| Cell area (μm^2^) | | **d** | **2x** | **4x** |
| --- | --- | --- | --- | --- |
| Xylem | | 25 d | 66.01±16.38c | 180.67±19.14b |
|  |  | 50 d | 72.98±10.51c | 196.46±36.44ab |
|  |  | 70 d | 63.92±27.86c | 236±28.83a |
|  | | 25 d | 510.55±26.38d | 647.95±37.82cd |
| Cortex | 50 d | | 705.6±51.65bcd | 1046.27±183.86b |
|  | | 70 d | 955.57±254.5bc | 1969.31±350.84a |
| Phloem | | 25 d | 37.33±21.4b | 49.92±3.01b |
|  |  | 50 d | 39.71±5.53b | 81.75±9.47a |
|  |  | 70 d | 53.55±13.09b | 82.85±4.51a |
| Pith | | 25 d | 818.92±14.55c | 1289.54±123.38ab |
|  |  | 50 d | 973.49±261.18bc | 1281.98±261.05ab |
|  |  | 70 d | 935.1±115.99bc | 1534.14±215.28a |

Note: Data are represented as the mean ± SE of three replicates. Different lowcase letters indicate significant differences among treatments as determined by Duncan’s test（*p* ≤ 0.05）.

**Table S7** Comparison of root anatomy between diploid and tetraploid of hybrid sweetgum

| Thickness (μm) | **d** | **2x** | **4x** |
| --- | --- | --- | --- |
| Diameter | 25 d | 349.37±35.63bc | 460.17±37.92bc |
|  | 50 d | 331.98±42.15c | 660.92±152.37a |
|  | 70 d | 489.32±44.77b | 721.34±80.48a |
| Epidermis | 25 d | 7.99±0.88b | 19.91±1.55a |
|  | 50 d | 10.24±1.59b | 20.52±5.06a |
|  | 70 d | 10.74±1.45b | 20.89±5.47a |
| Cortex | 25 d | 87.96±5.47c | 147.62±27.91b |
|  | 50 d | 92.67±13.36bc | 269.63±51.63a |
|  | 70 d | 122.01±10.81bc | 275.14±41.76a |
| Cylinder | 25 d | 120.57±10.4ab | 129.02±28.78ab |
|  | 50 d | 114.5±11.03b | 133.37±23.92ab |
|  | 70 d | 161.73±30.78a | 159.22±10.51a |

Note: Data are represented as the mean ± SE of three replicates. Different lowcase letters indicate significant differences among treatments as determined by Duncan’s test（*p* ≤ 0.05）.

**Table S8** Comparison of root cell characteristics between diploid and tetraploid hybrid sweetgum

| **Cell area** (um^2^) | **d** | **2x** | **4x** |
| --- | --- | --- | --- |
| Xylem | 25 d | 42.13±2.74c | 82.8±15.95ab |
|  | 50 d | 44.67±5.85bc | 93.69±34.67a |
|  | 70 d | 48.73±25.13bc | 96.57±21.43a |
| Epidermis | 25 d | 108.34±21.28c | 259.72±36.8b |
|  | 50 d | 115.54±27.36c | 368.26±103.66a |
|  | 70 d | 129.94±24.72c | 427.78±30.06a |
| Cortex | 25 d | 522.62±26.36c | 1429.04±222.22b |
|  | 50 d | 635.61±32.4c | 2313.67±420.59a |
|  | 70 d | 744.55±127.31c | 2658.82±495.11a |

Note: Data are represented as the mean ± SE of three replicates; Different lowcase letters indicate significant differences among treatments as determined by Duncan’s test（*p* ≤ 0.05）.

Table S9 The statistic of RNA-Seq data.

| **Sample** | **raw reads** | **raw**  **bases** | **clean reads** | **clean**  **bases** | **valid**  **bases** | **Q30** | **GC** |
| --- | --- | --- | --- | --- | --- | --- | --- |
| DR_1 | 49888822 | 7483323300 | 47540848 | 6859613847 | 91.67% | 94.24% | 46.23% |
| DR_2 | 49771960 | 7465794000 | 47677234 | 6895263850 | 92.36% | 94.62% | 46.23% |
| DR_3 | 49888790 | 7483318500 | 47589892 | 6863824060 | 91.72% | 94.45% | 46.65% |
| DS_1 | 45436364 | 6815454600 | 43526576 | 6255456636 | 91.78% | 94.62% | 46.61% |
| DS_2 | 49056170 | 7358425500 | 46752306 | 6732770965 | 91.50% | 94.35% | 46.39% |
| DS_3 | 49832960 | 7474944000 | 47379724 | 6828019573 | 91.35% | 94.13% | 46.31% |
| TR_1 | 49020402 | 7353060300 | 46721918 | 6665738470 | 90.65% | 94.39% | 46.98% |
| TR_2 | 49452426 | 7417863900 | 47104392 | 6717127202 | 90.55% | 94.27% | 46.85% |
| TR_3 | 49127780 | 7369167000 | 46854810 | 6713238106 | 91.10% | 94.27% | 46.68% |
| TS_1 | 49277478 | 7391621700 | 47582882 | 6848853323 | 92.66% | 95.07% | 46.60% |
| TS_2 | 49038682 | 7355802300 | 46834194 | 6758771831 | 91.88% | 94.47% | 46.14% |
| TS_3 | 49687090 | 7453063500 | 47538944 | 6857438520 | 92.01% | 93.45% | 46.37% |

Table S10 Statistics of assembled results

| **Term** | **>300bp** | **≥500bp** | **≥1000bp** | **N50** | **total length** | **Max** | **Mix** | **Mean** |
| --- | --- | --- | --- | --- | --- | --- | --- | --- |
| Unigene | 60187 | 35118 | 18857 | 1343 | 55200079 | 10802 | 301 | 917.14 |

**Table S11** Annotation of assembled unigenes against seven public databases.

| **Anno_Database** | **Annotated_Number** | **300<=length<1000** | **length>=1000** |
| --- | --- | --- | --- |
| NR | 38411 (63.82 %) | 20352 (33.81 %) | 18059 (30.00 %) |
| Swissprot | 28548 (47.43 %) | 13170 (21.88 %) | 15378 (25.55 %) |
| KEGG | 13096 (21.76 %) | 6164 (10.24 %) | 6932 (11.52 %) |
| KOG | 21237 (35.29 %) | 10201 (16.95 %) | 11036 (18.34 %) |
| eggNOG | 35419 (58.85 %) | 17746 (29.48 %) | 17673 (29.36 %) |
| GO | 24841 (41.27 %) | 11087 (18.42 %) | 13754 (22.85 %) |
| Pfam | 145 (0.24 %) | 133 (0.22 %) | 12 (0.02 %) |

**Table S12** statistical table of differential expressionUnigenes

| **Case** | **Control** | **Up_diff** | **Down_diff** | **Total_diff** |
| --- | --- | --- | --- | --- |
| TR1,TR2,TR3(TR) | DS1,DS2,DS3(DS) | 7367 | 7896 | 15263 |
| TR1,TR2,TR3(TR) | TS1,TS2,TS3(TS) | 6649 | 7190 | 13839 |
| TS1,TS2,TS3(TS) | DS1,DS2,DS3(DS) | 7425 | 6670 | 14095 |
| TR1,TR2,TR3(TR) | DR1,DR2,DR3(DR) | 4160 | 8957 | 13117 |
| DR1,DR2,DR3(DR) | DS1,DS2,DS3(DS) | 9144 | 4430 | 13574 |

Note：(*p* value<0.05&|log2FC|>1)

**Table S13** GO top up in shoot

| id | term | category |
| --- | --- | --- |
| GO:0046039 | GTP metabolic process | BP |
| GO:0038128 | ERBB2 signaling pathway | BP |
| GO:0045487 | gibberellin catabolic process | BP |
| GO:0015074 | DNA integration | BP |
| GO:0044550 | secondary metabolite biosynthetic process | BP |
| GO:0032197 | transposition, RNA-mediated | BP |
| GO:0005983 | starch catabolic process | BP |
| GO:0032199 | reverse transcription involved in RNA-mediated transposition | BP |
| GO:0009820 | alkaloid metabolic process | BP |
| GO:0006310 | DNA recombination | BP |
| GO:0031981 | nuclear lumen | CC |
| GO:0005925 | focal adhesion | CC |
| GO:0016602 | CCAAT-binding factor complex | CC |
| GO:0043661 | peribacteroid membrane | CC |
| GO:0071008 | U2-type post-mRNA release spliceosomal complex | CC |
| GO:0000943 | retrotransposon nucleocapsid | CC |
| GO:0070062 | extracellular exosome | CC |
| GO:0045277 | respiratory chain complex IV | CC |
| GO:0031090 | organelle membrane | CC |
| GO:0000221 | vacuolar proton-transporting V-type ATPase, V1 domain | CC |
| GO:0004411 | homogentisate 1,2-dioxygenase activity | MF |
| GO:0004157 | dihydropyrimidinase activity | MF |
| GO:0003964 | RNA-directed DNA polymerase activity | MF |
| GO:0004523 | RNA-DNA hybrid ribonuclease activity | MF |
| GO:0003676 | nucleic acid binding | MF |
| GO:0004497 | monooxygenase activity | MF |
| GO:0004190 | aspartic-type endopeptidase activity | MF |
| GO:0005506 | iron ion binding | MF |
| GO:0004519 | endonuclease activity | MF |
| GO:0016705 | oxidoreductase activity, acting on paired donors, with incorporation or reduction of molecular oxygen | MF |

**Table S14** GO top down in shoot

| id | term | category |
| --- | --- | --- |
| GO:0016572 | histone phosphorylation | BP |
| GO:0071490 | cellular response to far red light | BP |
| GO:0071491 | cellular response to red light | BP |
| GO:0097150 | neuronal stem cell population maintenance | BP |
| GO:0000729 | DNA double-strand break processing | BP |
| GO:0033567 | DNA replication, Okazaki fragment processing | BP |
| GO:0065004 | protein-DNA complex assembly | BP |
| GO:0052096 | formation by symbiont of syncytium involving giant cell for nutrient acquisition from host | BP |
| GO:0010342 | endosperm cellularization | BP |
| GO:0033274 | response to vitamin B2 | BP |
| GO:0000780 | condensed nuclear chromosome, centromeric region | CC |
| GO:0032133 | chromosome passenger complex | CC |
| GO:0000779 | condensed chromosome, centromeric region | CC |
| GO:0000796 | condensin complex | CC |
| GO:0036449 | microtubule minus-end | CC |
| GO:0042555 | MCM complex | CC |
| GO:0031436 | BRCA1-BARD1 complex | CC |
| GO:0005658 | alpha DNA polymerase:primase complex | CC |
| GO:0010005 | cortical microtubule, transverse to long axis | CC |
| GO:0005874 | microtubule | CC |
| GO:0035175 | histone kinase activity (H3-S10 specific) | MF |
| GO:0043139 | 5'-3' DNA helicase activity | MF |
| GO:0001882 | nucleoside binding | MF |
| GO:0035402 | histone kinase activity (H3-T11 specific) | MF |
| GO:0072354 | histone kinase activity (H3-T3 specific) | MF |
| GO:0008252 | nucleotidase activity | MF |
| GO:0016207 | 4-coumarate-CoA ligase activity | MF |
| GO:0004563 | beta-N-acetylhexosaminidase activity | MF |
| GO:0015929 | hexosaminidase activity | MF |
| GO:0102148 | N-acetyl-beta-D-galactosaminidase activity | MF |

**Table S15** GO top up in root

| id | term | category |
| --- | --- | --- |
| GO:0098755 | maintenance of seed dormancy by abscisic acid | BP |
| GO:0010258 | NADH dehydrogenase complex (plastoquinone) assembly | BP |
| GO:0009645 | response to low light intensity stimulus | BP |
| GO:0071497 | cellular response to freezing | BP |
| GO:0080136 | priming of cellular response to stress | BP |
| GO:0080169 | cellular response to boron-containing substance deprivation | BP |
| GO:0015979 | photosynthesis | BP |
| GO:0009768 | photosynthesis, light harvesting in photosystem I | BP |
| GO:0010411 | xyloglucan metabolic process | BP |
| GO:0009607 | response to biotic stimulus | BP |
| GO:0009535 | chloroplast thylakoid membrane | CC |
| GO:0048046 | apoplast | CC |
| GO:0009534 | chloroplast thylakoid | CC |
| GO:0009523 | photosystem II | CC |
| GO:0016021 | integral component of membrane | CC |
| GO:0009507 | chloroplast | CC |
| GO:0010287 | plastoglobule | CC |
| GO:0009522 | photosystem I | CC |
| GO:0009579 | thylakoid | CC |
| GO:0000325 | plant-type vacuole | CC |
| GO:0016630 | protochlorophyllide reductase activity | MF |
| GO:0016655 | oxidoreductase activity, acting on NAD(P)H, quinone or similar compound as acceptor | MF |
| GO:0047100 | glyceraldehyde-3-phosphate dehydrogenase (NADP+) (phosphorylating) activity | MF |
| GO:0090353 | polygalacturonase inhibitor activity | MF |
| GO:0004497 | monooxygenase activity | MF |
| GO:0020037 | heme binding | MF |
| GO:0016168 | chlorophyll binding | MF |
| GO:0016762 | xyloglucan:xyloglucosyl transferase activity | MF |
| GO:0005506 | iron ion binding | MF |
| GO:0031409 | pigment binding | MF |

**Table S16** GO top down in root

| id | term | category |
| --- | --- | --- |
| GO:0000921 | septin ring assembly | BP |
| GO:0007089 | traversing start control point of mitotic cell cycle | BP |
| GO:0071931 | positive regulation of transcription involved in G1/S transition of mitotic cell cycle | BP |
| GO:1905534 | positive regulation of leucine import across plasma membrane | BP |
| GO:1905589 | positive regulation of L-arginine import across plasma membrane | BP |
| GO:2000045 | regulation of G1/S transition of mitotic cell cycle | BP |
| GO:2000134 | negative regulation of G1/S transition of mitotic cell cycle | BP |
| GO:0097308 | cellular response to farnesol | BP |
| GO:0008202 | steroid metabolic process | BP |
| GO:0090548 | response to nitrate starvation | BP |
| GO:0032151 | mitotic septin complex | CC |
| GO:0036391 | medial cortex septin ring | CC |
| GO:0120104 | actomyosin contractile ring, proximal layer | CC |
| GO:0022624 | proteasome accessory complex | CC |
| GO:0035068 | micro-ribonucleoprotein complex | CC |
| GO:0070578 | RISC-loading complex | CC |
| GO:0005940 | septin ring | CC |
| GO:0090619 | meiotic spindle pole | CC |
| GO:0055038 | recycling endosome membrane | CC |
| GO:0009337 | sulfite reductase complex (NADPH) | CC |
| GO:0070573 | metallodipeptidase activity | MF |
| GO:0000257 | nitrilase activity | MF |
| GO:0080061 | indole-3-acetonitrile nitrilase activity | MF |
| GO:0017046 | peptide hormone binding | MF |
| GO:0070330 | aromatase activity | MF |
| GO:0045503 | dynein light chain binding | MF |
| GO:0061631 | ubiquitin conjugating enzyme activity | MF |
| GO:0042282 | hydroxymethylglutaryl-CoA reductase activity | MF |
| GO:0047998 | hyoscyamine (6S)-dioxygenase activity | MF |
| GO:0004783 | sulfite reductase (NADPH) activity | MF |

**Table S17** KEGG enrichment analysis of DEGs between TS vs DS

| id | term | P val |
| --- | --- | --- |
| ko04080 | Neuroactive ligand-receptor interaction | 0 |
| ko00966 | Glucosinolate biosynthesis | 0 |
| ko00254 | Aflatoxin biosynthesis | 0 |
| ko00940 | Phenylpropanoid biosynthesis | 6.23E-11 |
| ko00500 | Starch and sucrose metabolism | 4.45E-09 |
| ko00520 | Amino sugar and nucleotide sugar metabolism | 5.47E-07 |
| ko04110 | Cell cycle | 1.20E-05 |
| ko04540 | Gap junction | 3.38E-05 |
| ko03030 | DNA replication | 3.39E-05 |
| ko00941 | Flavonoid biosynthesis | 6.17E-05 |
| ko00040 | Pentose and glucuronate interconversions | 6.87E-05 |
| ko04111 | Cell cycle - yeast | 7.04E-05 |
| ko00062 | Fatty acid elongation | 0.000316 |
| ko00791 | Atrazine degradation | 0.000374 |
| ko04075 | Plant hormone signal transduction | 0.000464 |
| ko00904 | Diterpenoid biosynthesis | 0.000502 |
| ko00130 | Ubiquinone and other terpenoid-quinone biosynthesis | 0.000595 |
| ko04113 | Meiosis - yeast | 0.001171 |
| ko00944 | Flavone and flavonol biosynthesis | 0.001858 |
| ko00905 | Brassinosteroid biosynthesis | 0.003222 |
| ko00400 | Phenylalanine, tyrosine and tryptophan biosynthesis | 0.003796 |
| ko00591 | Linoleic acid metabolism | 0.004554 |
| ko00052 | Galactose metabolism | 0.005046 |
| ko00270 | Cysteine and methionine metabolism | 0.005862 |
| ko00360 | Phenylalanine metabolism | 0.00635 |
| ko00604 | Glycosphingolipid biosynthesis - ganglio series | 0.007575 |
| ko00523 | Polyketide sugar unit biosynthesis | 0.007575 |
| ko00240 | Pyrimidine metabolism | 0.008415 |
| ko00053 | Ascorbate and aldarate metabolism | 0.008948 |
| ko00010 | Glycolysis / Gluconeogenesis | 0.010186 |
| ko01220 | Degradation of aromatic compounds | 0.011142 |
| ko00900 | Terpenoid backbone biosynthesis | 0.01403 |
| ko00906 | Carotenoid biosynthesis | 0.014642 |
| ko00643 | Styrene degradation | 0.018586 |
| ko00942 | Anthocyanin biosynthesis | 0.019007 |
| ko00982 | Drug metabolism - cytochrome P450 | 0.019294 |
| ko00460 | Cyanoamino acid metabolism | 0.019453 |
| ko00350 | Tyrosine metabolism | 0.022524 |
| ko00945 | Stilbenoid, diarylheptanoid and gingerol biosynthesis | 0.027089 |
| ko00603 | Glycosphingolipid biosynthesis - globo series | 0.027771 |
| ko04151 | PI3K-Akt signaling pathway | 0.027905 |
| ko00524 | Neomycin, kanamycin and gentamicin biosynthesis | 0.029744 |
| ko00980 | Metabolism of xenobiotics by cytochrome P450 | 0.032416 |
| ko00521 | Streptomycin biosynthesis | 0.037205 |
| ko04370 | VEGF signaling pathway | 0.040185 |
| ko04514 | Cell adhesion molecules (CAMs) | 0.042633 |
| ko04210 | Apoptosis | 0.046102 |

**Table S18** KEGG enrichment analysis of DEGs between TS vs DS

| id | term | pval |
| --- | --- | --- |
| ko00120 | Primary bile acid biosynthesis | 0 |
| ko00940 | Phenylpropanoid biosynthesis | 2.50E-10 |
| ko00500 | Starch and sucrose metabolism | 8.09E-07 |
| ko00906 | Carotenoid biosynthesis | 1.04E-06 |
| ko04540 | Gap junction | 1.34E-06 |
| ko02010 | ABC transporters | 3.47E-06 |
| ko00982 | Drug metabolism - cytochrome P450 | 3.91E-06 |
| ko00909 | Sesquiterpenoid and triterpenoid biosynthesis | 1.36E-05 |
| ko01212 | Fatty acid metabolism | 1.89E-05 |
| ko00980 | Metabolism of xenobiotics by cytochrome P450 | 3.44E-05 |
| ko04520 | Adherens junction | 4.06E-05 |
| ko00196 | Photosynthesis - antenna proteins | 7.15E-05 |
| ko04014 | Ras signaling pathway | 7.43E-05 |
| ko04015 | Rap1 signaling pathway | 8.60E-05 |
| ko00620 | Pyruvate metabolism | 9.94E-05 |
| ko04012 | ErbB signaling pathway | 0.000107 |
| ko04145 | Phagosome | 0.000252 |
| ko00061 | Fatty acid biosynthesis | 0.000261 |
| ko00904 | Diterpenoid biosynthesis | 0.000278 |
| ko04075 | Plant hormone signal transduction | 0.000278 |
| ko00480 | Glutathione metabolism | 0.000287 |
| ko04024 | cAMP signaling pathway | 0.000401 |
| ko00071 | Fatty acid degradation | 0.000402 |
| ko04510 | Focal adhesion | 0.00047 |
| ko04370 | VEGF signaling pathway | 0.000482 |
| ko01200 | Carbon metabolism | 0.000746 |
| ko00010 | Glycolysis / Gluconeogenesis | 0.000747 |
| ko00040 | Pentose and glucuronate interconversions | 0.001117 |
| ko00903 | Limonene and pinene degradation | 0.001157 |
| ko00625 | Chloroalkane and chloroalkene degradation | 0.001237 |
| ko00460 | Cyanoamino acid metabolism | 0.001446 |
| ko00195 | Photosynthesis | 0.001446 |
| ko00905 | Brassinosteroid biosynthesis | 0.00155 |
| ko00910 | Nitrogen metabolism | 0.002693 |
| ko04630 | Jak-STAT signaling pathway | 0.003248 |
| ko00710 | Carbon fixation in photosynthetic organisms | 0.003859 |
| ko04013 | MAPK signaling pathway - fly | 0.004227 |
| ko00073 | Cutin, suberine and wax biosynthesis | 0.005143 |
| ko00290 | Valine, leucine and isoleucine biosynthesis | 0.005495 |
| ko00350 | Tyrosine metabolism | 0.005946 |
| ko00100 | Steroid biosynthesis | 0.008155 |
| ko01040 | Biosynthesis of unsaturated fatty acids | 0.008199 |
| ko00253 | Tetracycline biosynthesis | 0.008647 |
| ko00561 | Glycerolipid metabolism | 0.008896 |
| ko00020 | Citrate cycle (TCA cycle) | 0.011166 |
| ko00660 | C5-Branched dibasic acid metabolism | 0.011899 |
| ko04011 | MAPK signaling pathway - yeast | 0.014066 |
| ko00270 | Cysteine and methionine metabolism | 0.015239 |
| ko04550 | Signaling pathways regulating pluripotency of stem cells | 0.016204 |
| ko00730 | Thiamine metabolism | 0.016287 |
| ko00531 | Glycosaminoglycan degradation | 0.016287 |
| ko04310 | Wnt signaling pathway | 0.016383 |
| ko01053 | Biosynthesis of siderophore group nonribosomal peptides | 0.016755 |
| ko00920 | Sulfur metabolism | 0.016842 |
| ko00361 | Chlorocyclohexane and chlorobenzene degradation | 0.017041 |
| ko00364 | Fluorobenzoate degradation | 0.017041 |
| ko00623 | Toluene degradation | 0.017041 |
| ko04066 | HIF-1 signaling pathway | 0.020373 |
| ko00514 | Other types of O-glycan biosynthesis | 0.022088 |
| ko00062 | Fatty acid elongation | 0.025448 |
| ko00380 | Tryptophan metabolism | 0.031014 |
| ko00900 | Terpenoid backbone biosynthesis | 0.032713 |
| ko00330 | Arginine and proline metabolism | 0.03395 |
| ko04810 | Regulation of actin cytoskeleton | 0.039138 |
| ko00944 | Flavone and flavonol biosynthesis | 0.04078 |
| ko00471 | D-Glutamine and D-glutamate metabolism | 0.04078 |
| ko00401 | Novobiocin biosynthesis | 0.04078 |
| ko00942 | Anthocyanin biosynthesis | 0.04078 |
| ko00565 | Ether lipid metabolism | 0.041516 |
| ko04071 | Sphingolipid signaling pathway | 0.04159 |
| ko02020 | Two-component system | 0.045737 |
| ko00310 | Lysine degradation | 0.047466 |

**Table S19** Effects of hormone and medium replacement time on the elongation of tetraploid of hybrid sweetgum

|  | **0 d** | | **30 d** | | **40 d** | |
| --- | --- | --- | --- | --- | --- | --- |
| **Hormone types** | **Plant height** | **Root length** | **Plant height** | **Root length** | **Plant height** | **Root**  **length** |
| IAA | ﹢﹢ | ﹢﹢﹢ | ﹢ | ﹢﹢ | ﹢ | ﹢ |
| GA | ﹢﹢﹢ | ﹢﹢﹢ | ﹢﹢ | ﹢ | ﹢ | ﹢ |
| BR | ﹢ | ﹢ | ﹢ | ﹢ | ﹢ | ﹢ |

Note: “﹢﹢﹢”: good effective (30-50% longer than the control);“﹢﹢” effective(10-30% longer than the control); “+”: not very effective ( less 10% longer than the control).

**Table S20** Effects of IAA, GA_3_ and BR in different media on regenerated plantlet development of tetraploid hybrid sweetgum

| **No.** | **Plant height (cm)** | **Ground diameter (mm)** | **Internodes**  **number** | **leaf area**  **(mm^2^)** | **Root number** | **Root length**  **(mm)** |
| --- | --- | --- | --- | --- | --- | --- |
| 1 | 2.03±0.14d | 1.87±0.08e | 4.07±0.35b | 79.21±4.57c | 8.85±0.57cd | 7.1±0.22de |
| 2 | 2.9±0.73bcd | 2.32±0.1ab | 4.4±0.53ab | 99.07±4.01a | 12.55±0.67a | 10.47±0.17b |
| 3 | 4.42±0.97a | 2.45±0.05a | 4.8±0.26a | 91.18±4.3ab | 8.73±0.53cd | 10.45±1.22b |
| 4 | 2.06±0.19d | 1.91±0.03e | 4.1±0.2ab | 73.3±3.05c | 9.77±0.69bc | 6.51±0.35e |
| 5 | 4.02±1.04ab | 2.37±0.07ab | 4.7±0.44ab | 101.53±7.14b | 13.59±0.45a | 12.44±0.59a |
| 6 | 2.57±0.26cd | 2.02±0.17de | 4.27±0.4ab | 75.59±5.05c | 12.57±0.62a | 10.38±0.16b |
| 7 | 3.32±0.75abc | 2.26±0.05bc | 4.57±0.31ab | 81.09±11.47bc | 8.31±0.71d | 8.79±0.56c |
| 8 | 2.59±0.27cd | 2.14±0.04cd | 4.17±0.35ab | 72.18±4.3c | 10.53±0.59b | 7.84±0.47cd |

Note: 1-8 correspond to the medium composition of Table S2; Data are represented as the mean ± SE of three replicates; Different lowcase letters indicate significant differences among treatments as determined by Duncan’s test (*p* ≤ 0.05).
